# Bottle Gourd (*Lagenaria siceraria*): Novel Insights Into the Biochemical Propensities of the Unexplored Cultivar

**DOI:** 10.1155/sci5/7463939

**Published:** 2026-02-23

**Authors:** Hanan Y. Aati, Renad Al-Arifi, Chitchamai Ovatlarnporn, Mohsin Abbas Khan, Abdul Rauf, Hossam M. Hassan, Abdul Basit, Huma Rao, Laiba Rehman, Kashif ur Rehman Khan

**Affiliations:** ^1^ Department of Pharmacognosy, College of Pharmacy, King Saud University, P.O. Box 2457, Riyadh, 11451, Saudi Arabia, ksu.edu.sa; ^2^ College of Pharmacy, King Saud University, P.O. Box 2457, Riyadh, 11451, Saudi Arabia, ksu.edu.sa; ^3^ Department of Pharmaceutical Chemistry, Faculty of Pharmaceutical Sciences, Prince of Songkla University, Hat Yai, Songkhla, 90110, Thailand, psu.ac.th; ^4^ Drug Delivery System Excellence Center, Faculty of Pharmaceutical Sciences, Prince of Songkla University, Hat Yai, Songkhla, 90110, Thailand, psu.ac.th; ^5^ Department of Pharmaceutical Chemistry, Faculty of Pharmacy, The Islamia University of Bahawalpur, Bahawalpur, 63100, Punjab, Pakistan, iub.edu.pk; ^6^ Department of Pharmacognosy, College of Pharmacy, University of Kut, Wasit, 52001, Iraq; ^7^ Department of Pharmacognosy, Faculty of Pharmacy, Beni-Suef University, Beni-Suef, Egypt, bsu.edu.eg; ^8^ Department of Medicinal Chemistry, College of Pharmacy Shenzhen Technology University, Shenzhen, 518118, Guangdong, China; ^9^ College of Pharmacy, The University of Health Sciences, Lahore, 54600, Punjab, Pakistan

**Keywords:** anti-bacterial, antioxidant, GC-MS, *Lagenaria siceraria*, molecular docking, thrombolytic

## Abstract

The fruit of *Lagenaria siceraria* (bottle gourd), widely consumed as a vegetable, possesses notable health benefits. This study investigated the chemical and biological profiles of the Saudi cultivar through quantitative bioactive analysis, GC‐MS characterization, and in vitro evaluation of antioxidant, antibacterial, and enzyme inhibition activities. The 80% hydroethanolic extract (HEELS) exhibited a rich phytochemical profile, with the highest total tannin content (97.66 ± 3.33 mg/g). GC‐MS identified 55 compounds, including fatty acids, esters, sterols, and triterpenoids. HEELS displayed potent antioxidant activity, particularly in the FRAP assay (582 ± 1.18 mg AAE/g DE), and strong urease inhibition (91.8 ± 0.45%). Antibacterial assays showed the highest activity (12‐mm inhibition zone) against *Staphylococcus epidermidis* at 150 mg/mL. Molecular docking revealed significant interactions with α‐amylase, urease, and tyrosinase. These results suggest HEELS as a potential therapeutic agent, warranting further pharmacological and toxicological evaluation to confirm safety and efficacy for clinical applications.

## 1. Introduction

Natural origin‐based remedies have been used for hundreds of years for the treatment and eradication of many diseases. Herbal therapies are a good substitute for synthetic medications that are used at present, as they have minimal side effects and are effective in treating health ailments [1]. The medicinal food plants not only serve as a therapeutic alternative but also offer a preventive guard against different types of diseases. There are a large number of plants that have been said to possess therapeutic and preventive properties against antidiabetic effects, infections, cancers, and impart immunomodulatory effects [2]. These days, a lot of medical professionals and academics are using antioxidant‐based therapies as a primary tactic to prevent or reverse the abnormal physiology [3]. The commonly used vegetables and fruits have a plethora of functionally active constituents that are responsible for the therapeutic effects of the species. *Lagenaria siceraria* is one of them, which has been greatly cultivated in various countries for food and therapeutic purposes due to its diverse pharmacological properties [4]. It is also referred to as bottle gourd (English) and Lauki (Hindi) and “Duba” in Arabic. It has recently been introduced in the Kingdom of Saudi Arabia for cultivation. Saudi Arabia hosts a wide variety of medicinal herbs, thanks to its distinctive geography and diverse climate that promote the growth of numerous plant species. *L. siceraria* is cultivated in various regions of Saudi Arabia, such as Jazan, where it flourishes under suitable conditions. This trailing herb is among the oldest plants on earth, having fruits that resemble bottles or dumbbells. The leaves have emetic, purgative properties and ameliorate baldness. Roots have emetic properties and are used in dropsy [5].

Traditionally, *L*. *siceraria* has been used in folk medicine to treat ailments such as fever, ulcers, and asthma and as a general tonic. *L*. *siceraria* has a high‐water content and low‐calorie count that make it a favored choice for both culinary and medicinal purposes. Phytochemicals such as vitamins, choline, proteins, minerals, sterols, flavonoids, cucurbitacins, triterpenoids, C‐glycosides, flavones, and β‐glycosides are present in it [1, 6]. The main constituents that have been identified and reported by researchers using GC‐MS from the fruit seed pulp were fatty acids and their esters [7]. Various parts of the species, fruit, root, flowers, and leaves have been assessed by researchers for pharmacological activities such as antidepressant, antianxiety, diuretic, cytotoxic, antimicrobial, antihyperlipidemic, analgesic, cardioprotective, anti‐inflammatory, anti‐hyperglycemic, anthelmintic, antihepatotoxic, antistress, antiurolithiatic, antiulcer, hepatoprotective, anticancer, immunomodulatory, and antioxidant effects [1].

*L. siceraria* has been reported for its potential antioxidant effects and enzyme inhibition activities. It is important to mention here that the antioxidant‐mediated therapeutic properties of the species contribute significantly to the attenuation of pathophysiological alterations of human biological systems. Similarly, enzymes serve as the vital drug targets, helping in the drug discovery and development. Various medicinal plants can inhibit clinically critical enzymes and address the respective disease state. To the best of our knowledge and literature search, the Saudi bottle gourd has not yet been studied for its chemical and biological profile. Therefore, in the present study, we focused on the chemical profiling of hydroethanolic (80%) extract of *L. siceraria* fruits *via* preliminary phytochemical identification, total bioactive content determination, and GC‐MS analysis, and a thorough biological investigation was carried out through antioxidant and enzyme inhibition assays (along with in silico studies) and antibacterial and thrombolytic assays to provide insights into chemical and biological propensities of the Saudi cultivar of the bottle gourd. The current project will open the gate for the nutraceutical and pharmaceutical industries for further research to develop nutraceuticals and pharmaceutical alternatives.

## 2. Materials and Methods

### 2.1. Chemicals

The following chemicals were used: ethanol, dimethyl sulfoxide (DMSO), sulfuric acid, α‐naphthol, absolute ethanol, copper sulfate heptahydrate (CuSO_4_·7H_2_O), potassium hydroxide, potassium sodium tartrate, sodium hydroxide, ninhydrin, picric acid, bismuth subnitrate, glacial acetic acid, aluminum chloride, potassium iodide, lead acetate, chloroform, ferric chloride, potassium ferrocyanide, quercetin, sodium nitrite, Folin–Ciocalteu (Fc) reagent, gallic acid, anhydrous sodium carbonate, tannic acid, ascorbic acid, sodium phosphate, ammonium molybdate, 1,1‐diphenyl‐2‐picrylhydrazyl (DPPH), 2,2′‐azino‐bis(3‐ethylbenzothiazoline‐6‐sulfonic acid) (ABTS), potassium permanganate, acetic acid, hydrochloric acid, 2,4,6‐tris(2‐pyridyl)‐s‐triazine (TPTZ), sulfanilamide, phosphoric acid, naphthylethylenediamine dihydrochloride, urea, potassium dihydrogen phosphate, phenol, sodium nitroprusside, sodium hypochlorite, thiourea, urease, sodium chloride, starch, iodine, dinitrosalicylic acid (DNSA), kojic acid, L‐DOPA, streptokinase, Triton X‐100, barium chloride, normal saline, nutrient broth, and Mueller–Hinton agar.

### 2.2. Collection and Extraction of Plant Material

Fresh fruits of *L. siceraria* were collected from a farm located in Jazan, Saudi Arabia, in June 2022. The plant was taxonomically identified by Dr. Rajakrishnan Rajagopal (College of Science, King Saud University) and deposited under Voucher Number KSU No. 10579. The fruits were thoroughly washed, sliced into small pieces, and air‐dried under shade. The dried material (500 g) was pulverized into a fine powder and macerated in 80% ethanol (250 mL) for 72 h with intermittent stirring. After filtration, the process was repeated several times to ensure maximum extraction. The combined filtrates were concentrated at 45°C using a rotary evaporator to yield a semisolid dark‐green residue, which was stored in airtight amber vials at 4°C until further analysis.

### 2.3. Phytochemical Analysis

#### 2.3.1. Preliminary Phytochemical Determination

The HEELS extract was subjected to preliminary phytochemical screening to detect the presence of primary and secondary plant metabolites, including lipids, carbohydrates, proteins, alkaloids, phenols, tannins, amino acids, steroids, flavonoids, glycosides, and saponins [8].

#### 2.3.2. Total Bioactive Content Estimation

The total phenolic content (TPC) of HEELS was determined using a modified Fc method as described in the literature [9]. The TPC was expressed as milligrams of gallic acid equivalent (mg GAE) per gram of dried extract. Similarly, the total flavonoid content (TFC) was evaluated following a slightly modified version of the method reported in the literature [10]. The TFC was represented as milligrams of quercetin equivalent (mg QE) per gram of dried extract. The total tannin 3,5‐dinitrosalicylicntified using the Fc method [10]. TTC was expressed as milligrams of tannic acid equivalent (mg TAE) per gram of dried extract.

#### 2.3.3. Gas Chromatography‐Mass Spectrometry (GC‐MS) Analysis

The chemical profiling of the 80% hydroethanolic extract (HEELS) was performed using an Agilent 5977A GC system coupled with a 5977A Series Mass Selective Detector and an HP‐5MS capillary column (30 m × 0.25 mm × 0.25 μm). Helium (99.99% purity) served as the carrier gas at a constant flow rate of 1.2 mL/min. The injection port, transfer line, and ion source were maintained at 310°C, with ionization energy set at 70 eV. The oven temperature was programmed to increase from 60°C (held for 7 min) to 310°C at a rate of 5°C/min. A 1‐μL sample was injected in the split mode (50:1). Mass spectra were obtained in the range of 35–650 m/z. Tentative compound identification was achieved by comparing mass spectral fragmentation patterns and retention indices with those in the NIST 17.1 library using Agilent MassHunter software, as previously described by the reported method [10] with minor modifications.

### 2.4. Biological Activities

#### 2.4.1. Antioxidant Activity

##### 2.4.1.1. Free Radical Scavenging Assays

###### 2.4.1.1.1. DPPH Assay

Free radical scavenging activity was determined using the DPPH method, with slight modifications of the procedure reported in the literature [10]. Serial dilutions of the extract (15–50 μg/mL) were prepared in methanol. To each well of a microplate, 90 μL of sample solution was mixed with 90 μL of 0.3 mM DPPH solution and incubated in darkness for 30 min at room temperature. Absorbance was recorded at 515 nm, and ascorbic acid served as the standard. The results were expressed as mg ascorbic acid equivalents per g of dry extract.

###### 2.4.1.1.2. ABTS Assay

ABTS radical cation was generated by reacting 2.5 mM ABTS with 2.45 mM potassium permanganate in 5% DMSO and incubating for 30 min in darkness. Varying concentrations of the extract were mixed with the reagent solution and incubated for 10 min. The absorbance was measured at 515 nm, and antioxidant capacity was calculated as mg ascorbic acid equivalents per g dry extract [11].

###### 2.4.1.1.3. Nitric Oxide Scavenging (NOS) Assay

NOS potential was analyzed following a modified procedure from the literature [12]. A mixture of 1 mL of extract (1 mg/mL) and 0.25 mL of 25 mM sodium nitroprusside was incubated at 37°C for 2 h. After incubation, 0.5 mL of the reaction mixture was treated with 0.3 mL of Griess reagent and absorbance was measured at 570 nm.

##### 2.4.1.2. Total Antioxidant Activity Capacity (TAC) by Phosphomolybdenum Assay

TAC was determined by the following a modified procedure from the literature [11]. Each tube containing 130 μL of extract was mixed with 870 μL of reagent solution (0.6 M sulfuric acid, 28 mM sodium phosphate, and 4 mM ammonium molybdate). The mixture was incubated at 95°C for 90 min and cooled, and absorbance was measured at 515 nm. The results were expressed as mg ascorbic acid equivalents per g extract.

##### 2.4.1.3. Reducing Antioxidant Potential

###### 2.4.1.3.1. Ferric Reducing Antioxidant Power (FRAP) Assay

FRAP was estimated by the method described in Ref. [11] with some modifications. Briefly, 90 μL of FRAP reagent (acetate buffer, ferric chloride, and TPTZ) was added to 10 μL of extract solution and incubated for 30 min at room temperature. Absorbance was recorded at 593 nm, and the antioxidant activity was expressed as mg ascorbic acid equivalents per g dry extract.

#### 2.4.2. Enzyme Inhibition Assay

##### 2.4.2.1. Urease Enzyme Inhibition Assay

For urease, the method described by Ref. [13] was followed with a few changes. 0.025% urease in 5% DMSO (10 μL), 10 μL of 1 M phosphate (pH = 7), and 0.5 mg/mL extract solution made in 5% DMSO (10 μL) were placed into an ELIZA 96‐well microplate, and the plate was incubated at 37°C for 15 min. Afterward, substrate, that is, 2.25% aqueous urea (30 μL), was added, and the ELISA plates were once again incubated for 15 min at 37°C. Then, absorbance, that is, preread, was taken at 630 nm wavelength. Then, 30 μL phenol reagent (1% phenol + 0.005% sodium nitroprusside in distilled water) was added, to which 50 μL of alkali reagent (0.5% sodium hydroxide + 0.1% sodium hypochlorite) was added. Another 90 min of incubation at 37°C in a 96‐well microplate was granted. Again, absorbance, that is, postread, was measured at 630 nm wavelength. The standard utilized for this activity was thiourea, and methanol was taken as a negative control. The following formula was used to express the percentage urease inhibition of the extract solution:

(1) inhibition % of urease=100−Abs. of postread−Abs. of prereadAbs. of control×100.

##### 2.4.2.2. α‐Amylase Inhibition Assay

The α‐amylase inhibition activity was performed by using the DNSA method with some modifications. 100 μL of 0.02 M sodium phosphate buffer + 100 μL of 1 M sodium chloride + 100 μL of 1% soluble starch solution + 100 μL of plant extracts/standard acarbose/negative control methanol were added and incubated for 5 min at 37°C and followed by the addition of 195 μL of salivary amylase (collected from the saliva of a healthy individual and centrifuged at 4000 rpm). Again, the reaction mixture was incubated further for 10 min at a similar temperature, followed by the addition of 260 μL reaction terminator dinitrosalicylic acid. In the final step, 100 μL of iodine reagent (mixture of 1 g iodine + 1 g potassium iodide in 20 mL distilled water) was added, and absorbance was measured at 620 nm [14]. Percentage enzyme inhibitions were calculated by using the following formula:

(2) inhibition %=absorbance of control−absorbance of extractabsorbance of control×100.

##### 2.4.2.3. Tyrosinase Inhibition Assay

Tyrosinase inhibition activity was ascertained by employing L‐DOPA as a substrate using the modified dopachrome method [11]. For this, a 300 units/mL tyrosinase enzyme solution was made to which 10‐μL plant extract was added, followed by the addition of 150 μL phosphate buffer (pH 6.8). The microtiter plate was then incubated for 10 min, and observation was taken as preread at 480 nm. Then, 20 μL of levodopa (2 mM) was added, and the plate was incubated again at room temperature for 30 min, and again, reading was taken at 480 nm as postread. Kojic acid was used as a positive control.

#### 2.4.3. Thrombolytic Activity

The thrombolytic activity was performed by following the method described in Ref. [13] with some changes. 5 mL of blood was drawn from a healthy volunteer without having any history of anticoagulants or oral contraceptives, added to a centrifugation tube, and centrifuged for 15 min. After blood clot formation, serum was removed from the centrifugation tube without damaging the blood clot. The empty Eppendorf tube was weighed and incubated at 37°C for 45 min 0.5 mL of the blood clot was placed in an Eppendorf tube and weighed again. The weight of the blood clot was determined by subtracting the weight of the Eppendorf tube containing the blood clot from the weight of the empty Eppendorf tube and is considered the weight of the clot before lysis. 100 μL of plant extract solutions/standard streptokinase (prepared by the dilution of commercially available streptokinase [1,500,000 I.U.] injection with 5 mL of sterilized water)/negative control distilled water was added to an Eppendorf tube containing blood clot and incubated for 90 min at 37°C. After incubation, remove the supernatant and determine the weight of chloride after lysis by subtracting it from the weight of an empty Eppendorf tube. The percentage of clot lysis was determined by this formula:

(3) % clot lysis=weight of clot after lysisweight of clot before lysis×100.

#### 2.4.4. Hemolytic Activity

The hemolytic activity was performed as per the literature with several modifications [12]. Human RBCs were used to assess the preliminary toxicity of phytochemicals present in plant extract. 10 mL of human blood was taken from volunteers and poured into an ethylenediamine tetraacetic acid (EDTA) tube and centrifuged at 4000 rpm for 10 min. The top plasma layer was separated, and the remaining RBC layer was washed three times with phosphate‐buffered saline (PBS) having a pH of 7.4. Then, the washed cells were suspended in PBS. 975 μL of sample solution (1 mg/mL in methanol) and 25 μL of erythrocytes were taken in an Eppendorf tube and incubated at 37°C for 90 min, and centrifuged at 2000 rpm. The hemolysis (%) was estimated by assessing the absorbance of hemoglobin in the supernatant at the wavelength of 540 nm by using a BioTek Synergy HT microplate reader. 0.1% Triton X‐100 was used as a positive control, and PBS was used as a negative control. % hemolysis was calculated by the following formula:

(4) hemolysis %=absorption of sample−absorbance of negative controlabsorbance of positive control×100.

#### 2.4.5. Antimicrobial Activity

##### 2.4.5.1. Bacterial Strains

Bacterial strains were acquired from the Microbiology Lab of the Islamia University of Bahawalpur, Pakistan. These stains include one gram‐negative, that is, *Escherichia coli,* and two gram‐positive bacteria, including *Micrococcus luteus and Staphylococcus epidermidis*.

##### 2.4.5.2. Agar Well‐Diffusion Method

In this assay, the dried plant extracts were dissolved in methanol, and solutions of varying concentrations (50, 100, and 150 mg/mL) were prepared. The antimicrobial assay was performed by the disc diffusion method using 100 μL of the suspension having a bacterial cell density of 10^8^ CFU/mL on Mueller–Hinton agar medium. Filter paper discs, measuring 5 mm in diameter, were immersed in extract solutions and positioned on inoculated agar. Ciprofloxacin 1 mg/mL was applied as a standard antibacterial drug in studies. The petri dishes were incubated at 37°C for 24 h. Antibacterial effect was determined by measuring the zone of inhibition against the test organisms [15].

### 2.5. In Silico Studies

#### 2.5.1. Molecular Docking

Molecular docking analysis was carried out using PyRx software integrated with AutoDock Vina. Crystal structures of urease (PDB ID: 2Kau), tyrosinase (PDB ID: 2Y9X), and α‐amylase (PDB ID: 1BLI) were retrieved from the Protein Data Bank. The proteins were prepared by removing water molecules and hetatoms, followed by the addition of polar hydrogens using Discovery Studio 2021. Ligands identified via GC‐MS were downloaded from the PubChem database and converted from SDF to PDB format using Open Babel. Docking parameters were kept at exhaustiveness = 8, generating nine binding modes for each ligand. The best binding conformations were selected based on binding affinity, and interactions were visualized with Discovery Studio Visualizer [10].

#### 2.5.2. ADME Analysis

ADME analysis of the chosen bioactive compounds, chloride, with the highest binding affinity, was carried out by using the Swiss ADME online software (http://www.swissadme.ch/) [16].

#### 2.5.3. Toxicity Evaluation

The toxicity of the phytocompounds was virtually determined using the online program proTox‐3.0 (https://tox.charite.de/). [17].

### 2.6. Statistical Analysis

Readings in each experiment were taken in triplicate and expressed as mean ± standard deviation. One‐way ANOVA followed by Tukey’s multiple comparison test was applied to determine statistical significance using GraphPad Prism 7.0 software. The *p* value < 0.05 was considered statistically significant.

## 3. Results

### 3.1. Phytochemical Analysis

#### 3.1.1. Preliminary Phytochemical Screening

The phytoconstituents of HEELS were determined by employing qualitative phytochemical screening tests, which revealed the presence of primary and secondary metabolites from various classes, including carbohydrates, cardiac glycosides, flavonoids, phenols, tannins, and alkaloids. Table 1 shows the primary and secondary metabolites identified in ethanolic extracts of HEELS.

**Table 1:** Preliminary phytochemical screening of HEELS.

Metabolite	Test	Results
Amino acids	Ninhydrin test	−
Carbohydrates	Molisch’s reagent	+
Lipids	Saponification test	−
Protein	Biuret test	−
Reducing sugar	Fehling’s test	−
Cardiac glycosides	Keller Kiliani test	+
Flavonoids	Ferric chloride test	+
Phenols	Ferric chloride test	+
Saponins	Froth test	−
Steroids	Salkowski’s test	−
Tannins	Lead acetate test	+
Alkaloids	Hager’s test	+

*Note:* (+) Present and (−) absent.

#### 3.1.2. Total Bioactive Contents

The results of total bioactive contents estimation in HEELS are shown in Table 2. The TPC, TFC, and TTC of HEELS were calculated from a regression equation that was constructed from a gallic acid, quercetin, and tannic acid standard curve, respectively. The HEELS showed a TPC of 50.59 ± 0.36 mg GAE/g and a TFC of 38.11 ± 1.57 QE/g. TTC was found to be greater than TPC and TFC, having a value of 97.16 ± 3.11 TAE/g of dried extract.

**Table 2:** Total bioactive contents in HEELS.

Sample	TPC (mg GAE/g D.E)	TFC (mg QuE/g D.E)	TTC (mg TAE/g D.E)
HEELS	50.59 ± 0.36	38.11 ± 1.57	97.16 ± 3.11

*Note:* All tests were performed in triplicate, and the results are expressed as mean ± S.D.

#### 3.1.3. GC‐MS Analysis

**Figure 1:**
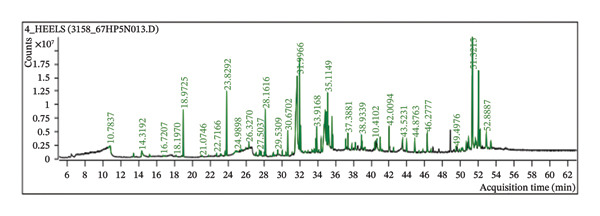
GC‐MS chromatogram of HEELS.

Following a thorough phytochemical characterization process, GC‐MS was used to further assess the chemical profile of HEELS. The analysis revealed HEELS with tentative identification of a total of 55 phytoconstituents. The compounds were identified by using the compounds’ peak retention duration, height (percent), and mass spectrum fragmentation patterns with those of recognized compounds present in NIST 17.1. Compounds identified through GC‐MS are given in Table 3, and their chromatogram is presented in Figure 1.

**Table 3:** Details of compounds identified in HEELS by the GC‐MS analyses.

Peak no	Retention time	Area (%)	Compound name	Molecular formula	Molecular weight	Chemical class
1	14.31	1.00	Coumaran	C_8_H_8_O	120.15	Benzo furan
2	15.20	0.19	2‐Decenal (E)‐	C_10_H_18_O	154.25	Aldehyde
3	16.72	0.14	2‐Methoxy‐4‐vinylphenol	C_9_H_10_O	150.17	Phenol
4	18.76	0.04	1‐Hexadecanol	C_16_H_34_O	242.44	Fatty alcohol
5	21.07	0.04	3‐Furanacetic acid, 4‐hexyl‐2,5‐dihydro‐2,5‐dioxo‐	C_12_H_16_O_5_	240.25	Cyclic dicarboxylic anhydride
6	21.81	0.05	Phenol, 2,4‐bis(1,1‐dimethylethyl)‐	C_14_H_22_O_5_	206.32	Phenol
7	22.71	0.11	Tetradecane, 2‐methyl‐	C_15_H_32_	212.41	Alkane
8	23.21	0.03	(‐)‐Spathulenol	C_15_H_24_O	220.35	Terpenoids
9	23.64	0.29	Diethyl phthalate	C_12_H_14_O_4_	222.24	Phthalate
10	24.98	0.43	Galacto‐heptulose	C_7_H_14_O_7_	210.18	Ketose sugar
11	25.5	0.04	1‐Hexadecanol, acetate	C_18_H_36_O_2_	284.5	Acetate ester
12	25.9	0.07	1,13‐Tridecanediol, diacetate	C_17_H_32_O_4_	300.4	Ester
13	27.14	0.15	Hexadecane, 2‐methyl‐	C_17_H_36_	240.5	Alkane
14	27.40	0.04	6‐Hydroxy‐4,4,7a‐trimethyl‐5,6,7,7a‐tetrahydrobenzofuran‐2(4H)‐one	C_11_H_32_O_4_	196.24	Benzo furan
15	27.50	0.55	Tetradecanoic acid	C_16_H_32_O_2_	228.37	Fatty acid
16	28.68	0.04	Pentadecanoic acid, methyl ester	C_16_H_32_O_2_	256.42	Fatty acid ester
17	28.94	0.03	Oleic acid	C_18_H_34_O_2_	282.5	Fatty acid
18	29.05	0.15	Phytol ketone	C_18_H_36_O	268.5	Ketone
19	29.22	0.06	Z‐11(13,13‐Dimethyl) tetradecen‐1‐ol acetate	C_18_H_34_O_2_	282.5	Acetylenic alcohols
20	30.44	0.15	7,9‐Di‐tert‐butyl‐1‐oxaspiro (4,5) deca‐6,9‐diene‐2,8‐dione	C_17_H_24_O_3_	276.4	α‐, β‐Unsaturated ketone
21	30.67	1.18	Hexadecanoic acid, methyl ester	C_17_H_34_O_2_	270.5	Fatty acid ester
22	31.72	15.8	n‐Hexadecanoic acid	C_16_H_32_O_2_	256.42	Fatty acid
23	31.99	4.72	Hexadecanoic acid, ethyl ester	C_18_H_36_O_2_	284.5	Fatty acid ester
24	32.30	0.07	1‐Eicosanol	C_20_H_42_O	298.5	Primary alcohol
25	32.93	0.11	Hexadecanoic acid, 2‐hydroxy‐, methyl ester	C_17_H_34_O_3_	286.4	Fatty acid ester
26	33.36	0.23	Heptadecanoic acid	C_17_H_34_O_2_	270.5	Fatty acid
27	33.61	0.21	1‐Octadecanol	C_18_H_38_O	270.5	Alcohol
28	33.78	0.76	9,12‐Octadecadienoic acid (Z,Z)‐, methyl ester	C_19_H_34_O_2_	294.5	Fatty acid ester
29	34.15	0.10	Phytol	C_20_H_40_O	296.5	Diterpene Alcohol
30	34.75	3.69	9,12‐Octadecadienoic acid (Z,Z)‐	C_18_H_32_O_2_	280.4	Unsaturated fatty acid
31	34.85	4.30	cis‐Vaccenic acid	C_18_H_34_O_2_	282.5	Unsaturated fatty acid
32	35.00	2.51	Linoleic acid ethyl ester	C_20_H_36_O_2_	308.5	Fatty acid ester
33	35.11	2.87	Ethyl oleate	C_20_H_38_O_2_	310.5	Ester
34	35.25	1.58	1‐Heptadecanecarboxylic acid	C_18_H_36_O_2_	284.5	Fatty acid
35	39.21	0.12	9‐Hydroxypentadecanoic acid, methyl ester	C_16_H_32_O_3_	272.4	Hydroxy ester
36	39.31	0.16	14b‐Pregnan	C_21_H_36_	288.5	Steroid
37	40.66	0.40	2‐Palmitoylglycerol	C_19_H_38_O_4_	330.5	Fatty acid ester
38	41.22	0.07	Bis(2‐ethylhexyl) phthalate	C_24_H_38_O_4_	390.6	Phthalate esters
39	42.00	1.19	Docosanoic acid, ethyl ester	C_24_H_48_O_2_	368.6	Fatty acid ester
40	46.70	0.16	Hexacosanoic acid, methyl ester	C_27_H_54_O_2_	410.7	Fatty acid ester
41	49.49	0.33	Dehydroergosterol	C_28_H_42_O	394.6	Phytosterols
42	49.86	0.19	Gamma‐sitosterol	C_27_H_30_O_15_	414.7	Phytosterols
43	50.10	0.17	5,6‐Dihydroergosterol	C_28_H_46_O	398.7	Phytosterols
44	50.23	0.20	Campesterol	C_28_H_48_O	400.7	Phytosterols
45	50.64	0.59	(22E)‐Stigmasta‐5,22‐dien‐3‐ol	C_29_H_48_O	412.7	Phytosterols
46	50.89	0.85	Ergost‐7‐en‐3‐ol (3‐β,5‐α)‐	C_28_H_48_O	400.7	Ergostanoid
47	51.04	0.12	9,19‐Cyclolanost‐7‐en‐3‐ol	C_30_H_50_O	426.7	Ergostanoid
48	51.32	9.18	Chondrillasterol	C_29_H_48_O	412.7	Steroid
49	51.65	0.82	β‐Amyrin	C_30_H_50_O	426.7	Pentacyclic terpenoids
50	51.74	0.68	Lanosterol	C_30_H_50_O	426.7	Tetracyclic terpenoids
51	52.00	4.97	5.α.‐Stigmast‐7‐en‐3.β.‐ol (24S)‐	C_29_H_50_O	414.7	Steroid
52	52.19	1.30	Lupeol	C_30_H_50_O	426.7	Pentacyclic terpenoids
53	52.88	1.18	Olean‐12‐en‐3‐one	C_30_H_48_O	424.7	Terpenoids
54	53.10	0.09	Toosendanin	C_30_H_38_O_11_	574.6	Terpenoids and saponins
55	53.39	0.60	Olean‐12‐EN‐3‐α‐YL acetate	C_32_H_52_O_2_	468.84	Terpenoids

### 3.2. Biological Activities

#### 3.2.1. Antioxidant Potential

The antioxidant capacity of HEELS was determined by DPPH, ABTS, NOS, TAC, and FRAP assays, and the results are shown in Table 4. The maximum antioxidant power is indicated by the FRAP assay (582.69 ± 1.12 mg AAE/g DE ± S.D). ABTS and NOS assays showed excellent antioxidant potential of 317.83 ± 2.71 mg AAE/g DE ± S.D. and 101.55 ± 1.92 mg AAE/g DE ± S.D., whereas TAC displayed good antioxidant power at 72.08 ± 1.22 mg AAE/g DE ± S.D.

**Table 4:** Antioxidant potential of HEELS.

Sample	ABTS	DPPH	FRAP	TAC	NOS
HEELS	317.83 ± 2.71	57.52 ± 0.07	582.69 ± 1.12	72.08 ± 1.22	101.55 ± 1.92

*Note:* All tests were performed in triplicate, and the results are expressed as mean ± S.D., mg of ascorbic acid equivalent per gram of dried extract.

#### 3.2.2. Enzyme Inhibition Assay

The enzyme inhibitory potential of HEELS was also investigated against three enzymes, urease, α‐amylase, and tyrosinase of clinical importance (Table 5). HEELS was found to be active against all enzymes. In urease activity, thiourea was employed as a positive standard to measure enzyme inhibition. The urease enzyme inhibition activity revealed that HEELS has a good inhibitory action of 91.78 ± 0.83%. However, α‐amylase showed an excellent enzyme inhibition of 58.92 ± 1.63%, and the standard used to perform this activity was acarbose. Tyrosinase activity also exhibited an acceptable enzyme inhibition potential of 81.64 ± 0.83% and the standard was kojic acid.

**Table 5:** Enzyme inhibitory activities of HEELS.

Extract/standard	Urease enzyme inhibition (%)	α‐Amylase inhibition (%)	Tyrosinase inhibition (%)
HEELS	91.78 ± 0.83	58.92 ± 1.63	81.64 ± 0.83
Standard	^a^94.20 ± 1.44	^b^96.04 ± 0.40	^c^88.40 ± 1.44

*Note:* All results are expressed as percentages and performed in triplicate.

^a^thiourea.

^b^acarbose.

^c^kojic acid.

#### 3.2.3. Thrombolytic Activity

The results of the thrombolytic assay of HEELS are displayed in Table 6. The % clot lysis of HEELS was 84.75 ± 1.22%, while streptokinase exhibited 98.97 ± 0.74% clot lysis.

**Table 6:** Thrombolytic and hemolytic assay of HEELS.

Extract/standard	Thrombolytic assay	Hemolytic assay
HEELS	84.75 ± 1.22	0.53 ± 0.15
Standard	^a^98.97 ± 0.74	^b^89.22 ± 0.58

*Note:* All results are expressed as percentages and performed in triplicate.

^a^streptokinase.

^b^Triton‐X.

#### 3.2.4. Hemolytic Activity

Table 6 represents the results of the hemolytic activity of HEELS. The % hemolytic assay value of the extract was 0.53 ± 0.15 compared to the standard of 89.22 ± 0.58%. As the extract value was found to be less than 30%, it is safe to be consumed as food [18].

#### 3.2.5. Antimicrobial Activity

**Figure 2:**
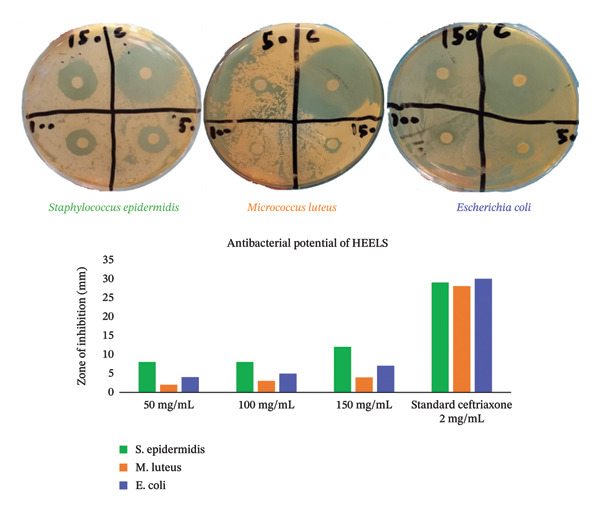
Antibacterial potential of HEELS. Results are represented in terms of mean diameter.

HEELS revealed the substantial activity of the extract in the assessment of its antibacterial properties. It has been proven that HEELS has dose‐dependent bactericidal action, as represented in Figure 2, and figures are shown in the supporting file as S1. The maximum activity was seen at 150 mg/mL. Furthermore, HEELS displayed a maximum bactericidal effect against *S. epidermidis* compared to the other two strains.

### 3.3. In Silico Studies

#### 3.3.1. Molecular Docking

*In silico* studies were done on all 55 compounds recognized by the GC‐MS analysis of HEELS to identify the binding affinity of bioactive compounds within the active pocket of targeted proteins (enzymes), that is, tyrosinase, urease, and α‐amylase. Before molecular docking investigations, active pocket residues of amino acids were determined through Discovery Studio 2021. The results are shown in Table 7. From the results, it was observed that β‐amyrin showed a maximum binding affinity for tyrosinase, whereas olean‐12‐en‐3‐one showed the highest affinity with urease enzyme. Lupeol showed the greatest binding affinity against α‐amylase. Twenty‐eight phytoconstituents obtained from GC‐MS analysis showed greater tyrosinase binding affinity as compared to standard kojic acid (binding energy, −5.3 kcal/mol). All 55 compounds showed greater urease binding potential than standard inhibitor thiourea (binding energy, −2.6 kcal/mol), while 16 bioactive constituents exhibited greater α‐amylase binding as compared to standard inhibitor acarbose (binding energy −7.7 kcal/mol).

**Table 7:** Binding affinity scores (kcal/mol) of phytoconstituents from HEELS’ GC‐MS.

Peak no	Compound name	Binding affinities tyrosinase	Binding affinities urease	Binding affinities α‐amylase
1	Coumaran	−6.2	−4.4	−5.4
2	2‐Decenal (E)‐	−5	−3.8	−4.6
3	2‐Methoxy‐4‐vinylphenol	−6.2	−4.6	−5.9
4	1‐Hexadecanol	−5	−4.3	−4.3
5	3‐Furanacetic acid, 4‐hexyl‐2,5‐dihydro‐2,5‐dioxo‐	−6.1	−5.4	−6.3
6	Phenol, 2,4‐bis(1,1‐dimethylethyl)‐	−6.6	−5.4	−6.3
7	Tetradecane, 2‐methyl‐	−5.1	−4	−4.5
8	(‐)‐Spathulenol	−7.9	−6	−6.5
9	Diethyl phthalate	−6.1	−4.9	−6
10	Galacto‐heptulose	−5.5	−4.4	−5.4
11	1‐Hexadecanol, acetate	−5	−4.3	−4.4
12	1,13‐Tridecanediol, diacetate	−5.1	−5.1	−4.9
13	Hexadecane, 2‐methyl‐	−5.2	−4.2	−4.5
14	6‐Hydroxy‐4,4,7a‐trimethyl‐5,6,7,7a‐tetrahydrobenzofuran‐2(4H)‐one	−7	−5.4	−6.8
15	Tetradecanoic acid	−4.6	−4.7	−5
16	Pentadecanoic acid, methyl ester	−5	−4.5	−4.4
17	Oleic acid	−4.6	−4.3	−5.2
18	Phytol ketone	−5.1	−4.9	−5.2
19	Z‐11(13,13‐Dimethyl) tetradecen‐1‐ol acetate	−5.2	−4.8	−5.1
20	7,9‐Di‐tert‐butyl‐1‐oxaspiro (4,5) deca‐6,9‐diene‐2,8‐dione	−6.6	−6.8	−7.3
21	Hexadecanoic acid, methyl ester	−4.7	−4.6	−4.4
22	n‐Hexadecanoic acid	−4.8	−4.5	−5.1
23	Hexadecanoic acid, ethyl ester	−4.8	−4.7	−5
24	1‐Eicosanol	−4	−4.4	−5.4
25	Hexadecanoic acid, 2‐hydroxy‐, methyl ester	−4.6	−5	−4.9
26	Heptadecanoic acid	−4.6	−4.5	−5.1
27	1‐Octadecanol	−5.5	−4.4	−4.8
28	9,12‐Octadecadienoic acid (Z,Z)‐, methyl ester	−5.2	−4.4	−5.1
29	Phytol	−4.6	−5	−5.1
30	9,12‐Octadecadienoic acid (Z,Z)‐	−5.3	−4.8	−5.1
31	cis‐Vaccenic acid	−5	−5	−5.1
32	Linoleic acid ethyl ester	−5	−4.4	−4.9
33	Ethyl oleate	−4.5	−4.8	−5
34	1‐Heptadecanecarboxylic acid	−4.7	−4.7	−5.1
35	9‐Hydroxypentadecanoic acid, methyl ester	−5	−4.6	−5.5
36	14b‐Pregnan	−7.7	−7.3	−8.1
37	2‐Palmitoylglycerol	−4.8	−4.9	−5.1
38	Bis(2‐ethylhexyl) phthalate	−5.5	−5.7	−5.7
39	Docosanoic acid, ethyl ester	−4	−5.2	−5.1
40	Hexacosanoic acid, methyl ester	−5.1	−4.5	−4.7
41	Dehydroergosterol	−8.2	−7.4	−8.9
42	Gamma sitosterol	−7.6	−6.6	−8
43	5,6‐Dihydroergosterol	−8.4	−7.3	−8.9
44	Campesterol	−7.7	−7.4	−8.3
45	(22E)‐Stigmasta‐5,22‐dien‐3‐ol	−8	−7.6	−8.7
46	Ergost‐7‐en‐3‐ol (3‐β,5‐α)‐	−8	−7	−8.6
47	9,19‐Cyclolanost‐7‐en‐3‐ol	−6.9	−7.1	−8.6
48	Chondrillasterol	−8.3	−7.4	−8.6
49	β‐Amyrin	−8.7	−8.4	−8.7
50	Lanosterol	−7.8	−7.3	−8.9
51	5‐α‐Stigmast‐7‐en‐3‐β‐ol (24S)‐	−7.8	−6.9	−8.2
52	Lupeol	−8.6	−7.4	−9.5
53	Olean‐12‐en‐3‐one	−7.9	−8.6	−9.3
54	Toosendanin	−7.4	−7.1	−8.3
55	Olean‐12‐EN‐3‐α‐YL acetate	−7.8	−7.6	−8.6
56	Standard	−5.3^a^	−2.6^b^	−7.7^c^

^a^kojic acid.

^b^Thiourea.

^c^Acarbose.

**Figure 3:**
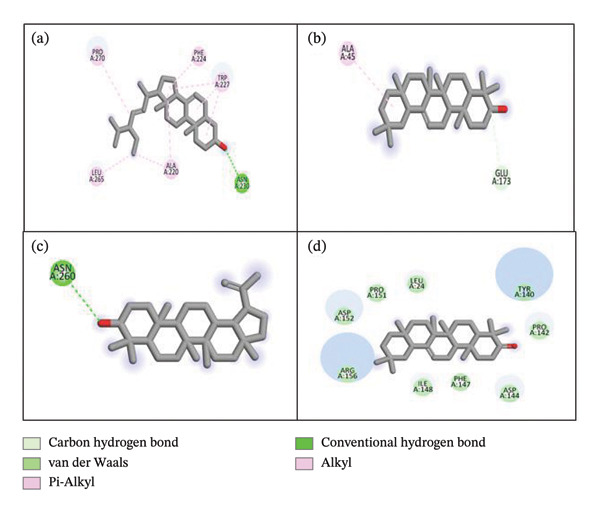
2D and 3D interaction of tyrosinase with (a) chondrillasterol, (b) β‐amyrin, (c) lupeol, and (d) olean‐12‐en‐3‐one.

**Figure 4:**
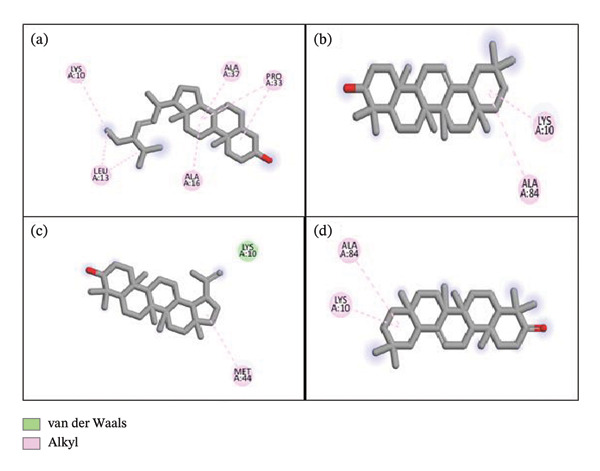
2D and 3D interaction of urease with (a) chondrillasterol, (b) β‐amyrin, (c) lupeol, and (d) olean‐12‐en‐3‐one.

**Figure 5:**
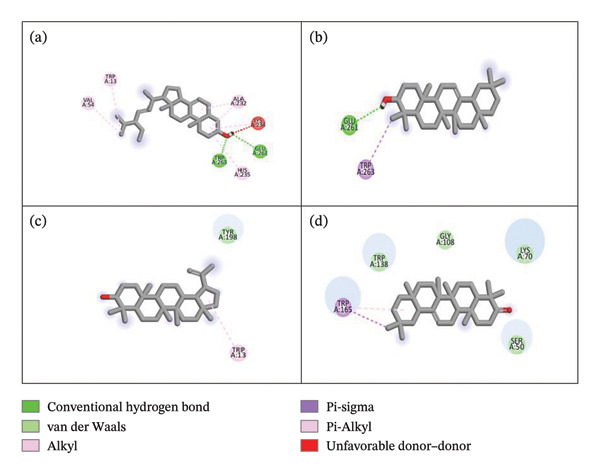
2‐D and 3D interactions of α‐amylase with (a) chondrillasterol, (b) β‐amyrin, (c) lupeol, and (d) olean‐12‐en‐3‐one.

The docking results suggested that out of 55 docked phytoconstituents, 16 compounds displayed maximum binding affinity as compared to the standard against all targeted enzymes. 2D interactions of ligands with tyrosinase, urease, and α‐amylase are shown in Figures 3, 4, and 5, respectively, while other compounds are presented in the supporting file as S2, S3, and S4, respectively. Chondrillasterol showed a conventional H bond with ASN 230, alkyl and pi–alkyl with PRO 270, LEU 265, ALA 220, PHE 224, and TRP 227. A carbon‐hydrogen bond was present between GLU 173 and an alkyl interaction between ALA 45 in the case of β‐amyrin. Lupeol displayed a C–H bond with ASN 260. Olean‐12‐en‐3‐one displayed van der Waals interaction with LEU 24, PRO 151, ASP 152, ARG 156, ILE 148, PHE 147, ASP 144, PRO 142, and TYR 140. However, it has been found that standard kojic acid displayed pi–sigma interaction with Val 283, pi–alkyl interaction with ALA 286. Pi–pi stacked and amide–pi stacked with HIS 263 SER 282.

Figure 4 represents some compounds with high docking affinity with the urease enzyme, while other compounds are presented in the supporting file as S3. Chondrillasterol displayed alkyl interaction with LYS 10, LEU 13, ALA 16, ALA 37, and PRO 33. Alkyl interaction with LYS 10 and ALA 84 has been observed for β‐amyrin. Lupeol exhibited van der Waals interaction with LYS 10 and MET 44. Olean‐12‐en‐3‐one showed alkyl interaction with ALA 84 and LYS 10. The standard thiourea was also docked against urease, and van der Waals interaction has been found between amino acids, PHE 14, ALA 84, MET 44, and ARG 48, and a conventional H bond is shown by GLU 45.

All 16 compounds with the best binding affinity were further analyzed for 2D and 3D interactions with α‐amylase. Figure 5 presents the four compounds with the best docking affinities, while other compounds are presented in the supporting file as S4. Chondrillasterol showed a conventional H bond with TRP 263 and GLU 261, an unfavorable donor–donor interaction with LYS 234, an alkyl and pi–alkyl interaction with LYS 234, VAL 54, TRP 13, HIS 235, and ALA 232. β‐Amyrin showed a conventional H bond with GLU 261and pi–sigma interaction with TRP 263. For lupeol, pi–alkyl and alkyl interactions are observed with TRP 13, and van der Waals interaction is observed with TYR 198. Olean‐12‐en‐3‐one exhibited a van der Waals interaction with TRP 138, GLY 108, LYS 70, SER 50, and pi–sigma and pi–alkyl with TRP 165. The standard acarbose exhibited a C–H bond with GLU 255, a conventional H bond with TYR 358, ASN 4, ALA 395, ARG 413, GLY 397, GLU 355, and an unfavorable acceptor–acceptor interaction with ASN 96.

#### 3.3.2. ADME Analysis

Ligands with the best docking results were subjected to ADME analysis by the online tool Swiss ADME. This tool gives information on drug kinetics, drug likeness, and physicochemical characteristics. In accordance with Lipinski’s rule, all compounds except toosendanin violated the rule in terms of lipophilicity. However, this ligand displayed three violations of Lipinski’s rule, which include hydrogen bond donor, molecular weight, and molecular refractivity. Gamma‐sitosterol (22E)‐stigmasta‐5,22‐dien‐3‐ol, 9,19‐cyclolanost‐7‐en‐3‐ol, chondrillasterol, β‐amyrin, lanosterol, 5‐α‐Stigmast‐7‐en‐3‐β‐ol (24S)‐, lupeol, olean‐12‐en‐3‐one, and olean‐12‐EN‐3‐α‐YL acetate violated two conditions: molar refractivity and lipophilicity. Drugs that do not fulfill two or more requirements are categorized as nonorally available drugs. However, ligands that exhibited a single violation may be recognized as orally bioavailable. Table 8 shows the ADME analysis, and Figure 6 shows the bioavailability radar of ligands having the best docking scores.

**Table 8:** Drug‐likeness prediction of selected ligands with high docking scores in HEELS.

Phytocompounds	Phytochemical properties				Lipophilicity	Lipinski’s rule
	HBD	HBA	MWT	MR		
14b‐Pregnan	0	0	288.51	94.09	7.21	Yes, 1 Vn
Dehydroergosterol	1	1	394.63	127.00	6.24	Yes, 1 Vn
Gamma‐sitosterol	1	1	414.71	133.23	6.73	Yes, 2 Vn
5,6‐Dihydroergosterol	1	1	398.66	127.95	6.43	Yes, 1 Vn
Campesterol	1	1	400.68	128.42	6.54	Yes, 1 Vn
(22E)‐Stigmasta‐5,22‐dien‐3‐ol	1	1	412.69	132.75	6.62	Yes, 2
Ergost‐7‐en‐3‐ol (3.β.,5.α.)‐	1	1	400.68	128.42	6.54	Yes, 1 Vn
9,19‐Cyclolanost‐7‐en‐3‐ol	1	1	426.72	135.14	6.92	Yes, 2 Vn
Chondrillasterol	1	1	412.69	132.75	6.62	Yes, 2 Vn
β‐Amyrin	1	1	426.72	134.88	6.92	Yes, 2 Vn
Lanosterol	1	1	426.72	137.04	6.82	Yes, 2 Vn
5.α.‐Stigmast‐7‐en‐3.β.‐ol (24S)‐	1	1	414.71	133.23	6.73	Yes, 2 Vn
Lupeol	1	1	426.72	135.14	6.92	Yes, 2 Vn
Olean‐12‐en‐3‐one	0	1	424.70	133.92	6.82	Yes, 2 Vn
Toosendanin	3	11	574.62	138.59	0.33	Yes. 3 Vn
Olean‐12‐EN‐3‐α‐YL acetate	0	2	468.75	144.62	7.08	Yes, 2 Vn

*Note:* MWT, molecular weight; Vn, violation.

Abbreviations: HBA, hydrogen bond acceptor; HBD, hydrogen bond donor, MR, molar refractivity.

Figure 6The bioavailability radar of ligands with the best docking scores. (a) 14‐b‐pregnan, (b) dehydroergosterol, (c) olean‐12‐EN‐3‐α‐YL acetate, (d) gamma‐sitosterol, (e) campesterol, (f) 5,6‐dihydroergosterol, (g) toosendanin, (h) (22E)‐stigmasta‐5,22‐dien‐3‐ol, (i) 9,19‐cyclolanost‐7‐en‐3‐ol, (j) chondrillasterol, (k) olean‐12‐en‐3‐one, (l) lanosterol, (m) ergost‐7‐en‐3‐ol, (3.β.,5.α.), (n) β‐amyrin, (o) lupeol, (p); 5.α.‐Stigmast‐7‐en‐3.β.‐ol (24S).

**Figure:**
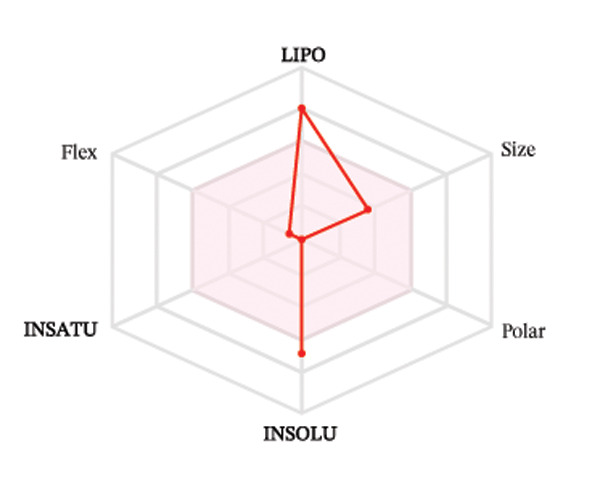
(a)

**Figure:**
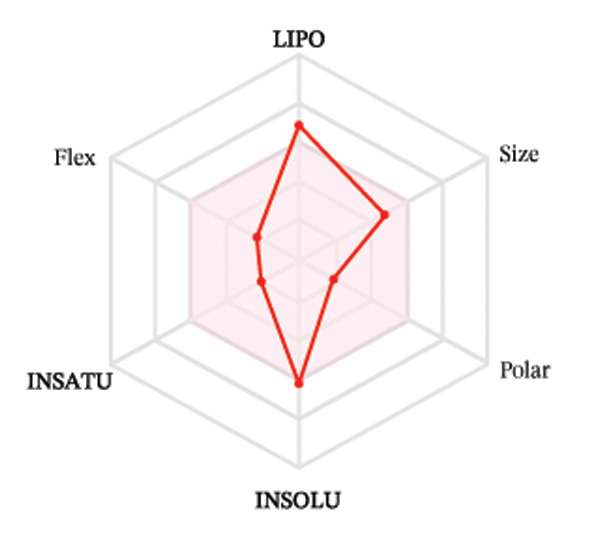
(b)

**Figure:**
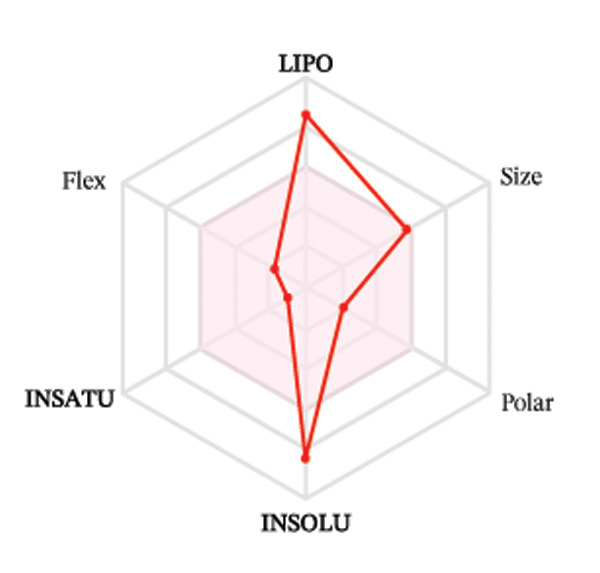
(c)

**Figure:**
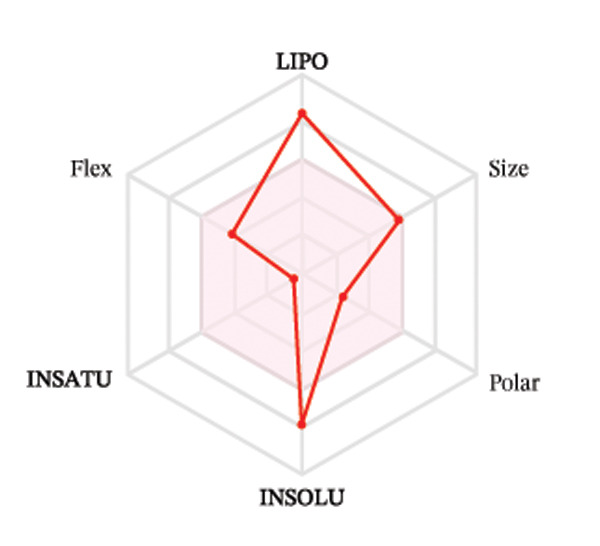
(d)

**Figure:**
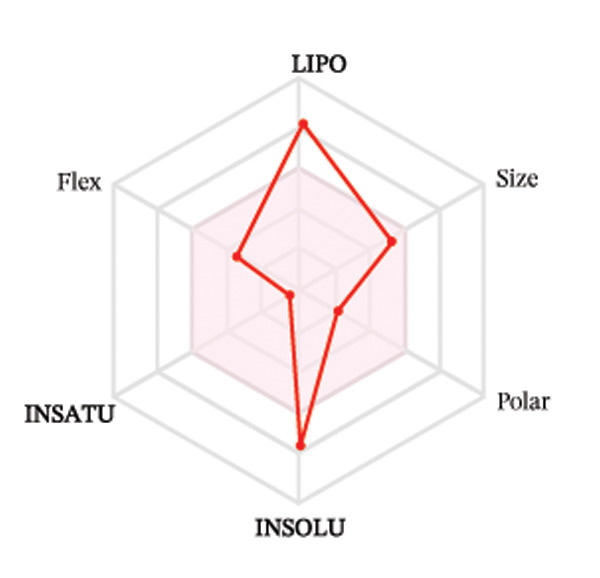
(e)

**Figure:**
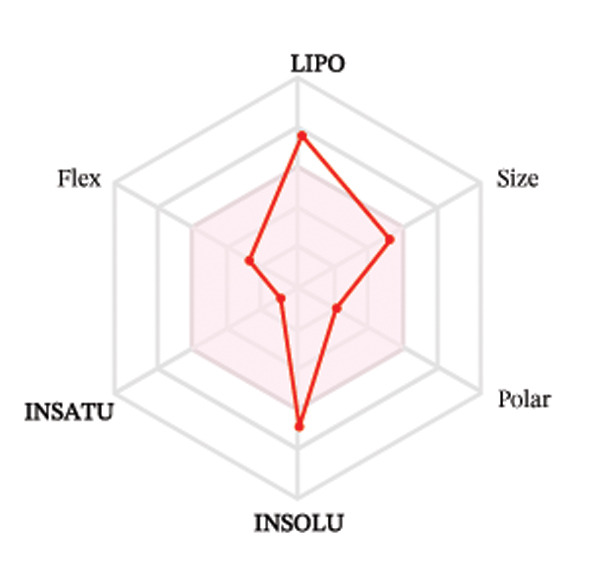
(f)

**Figure:**
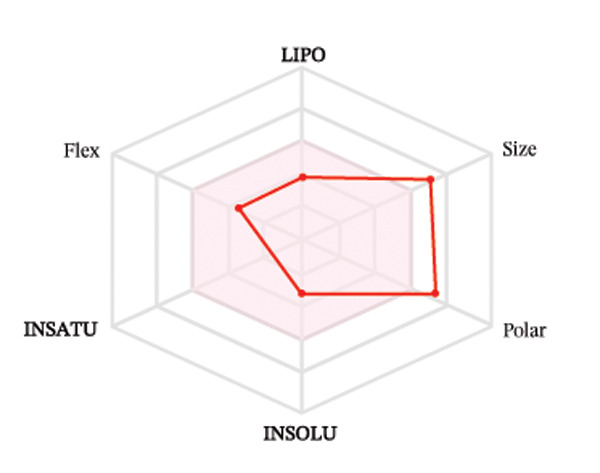
(g)

**Figure:**
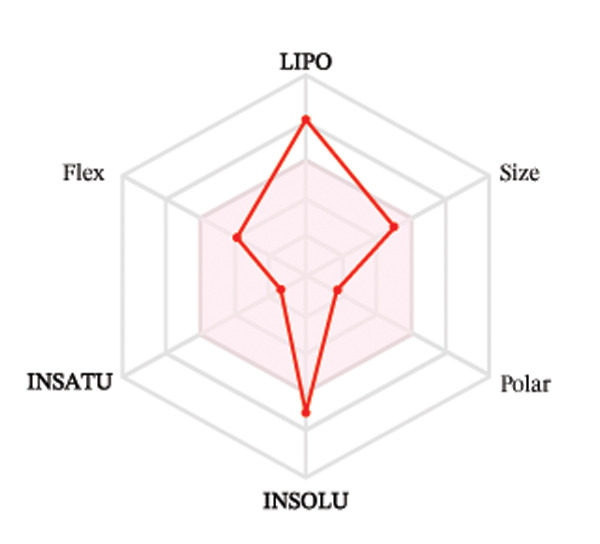
(h)

**Figure:**
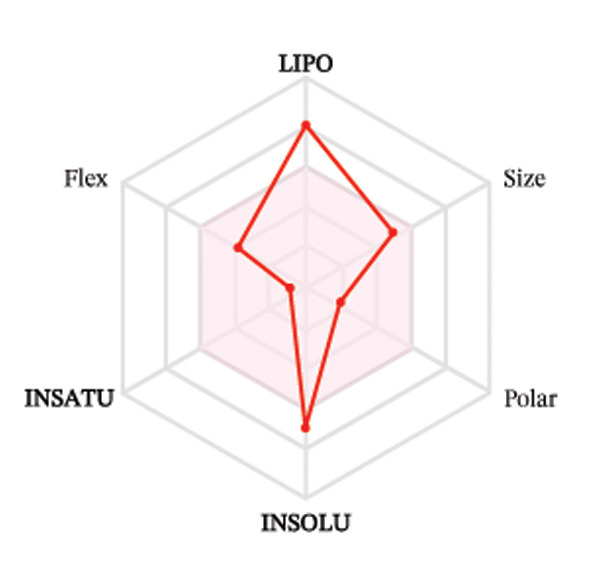
(i)

**Figure:**
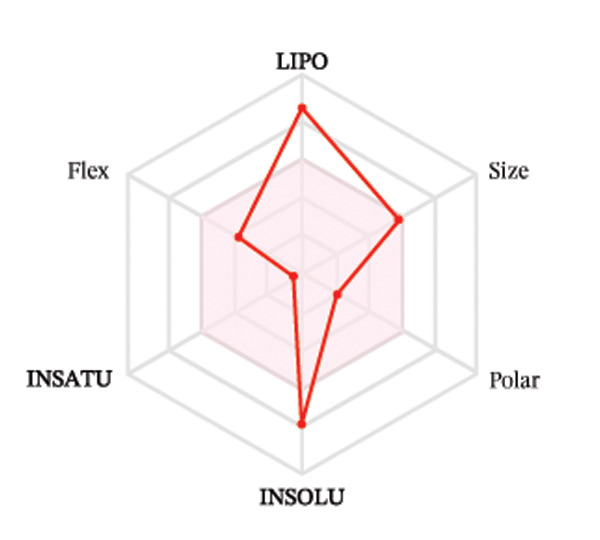
(j)

**Figure:**
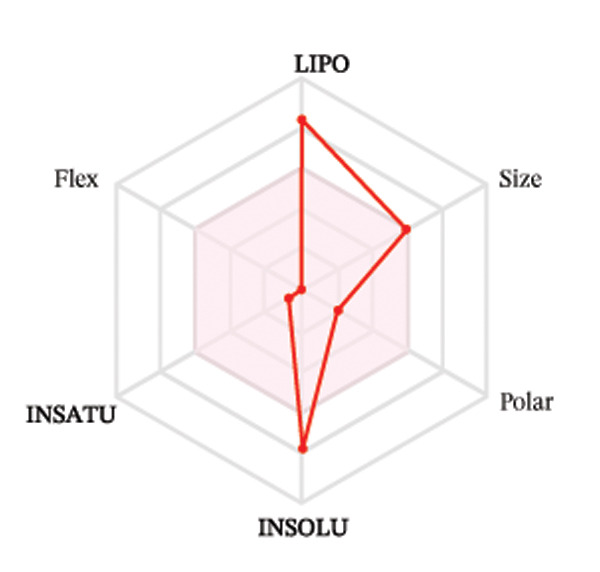
(k)

**Figure:**
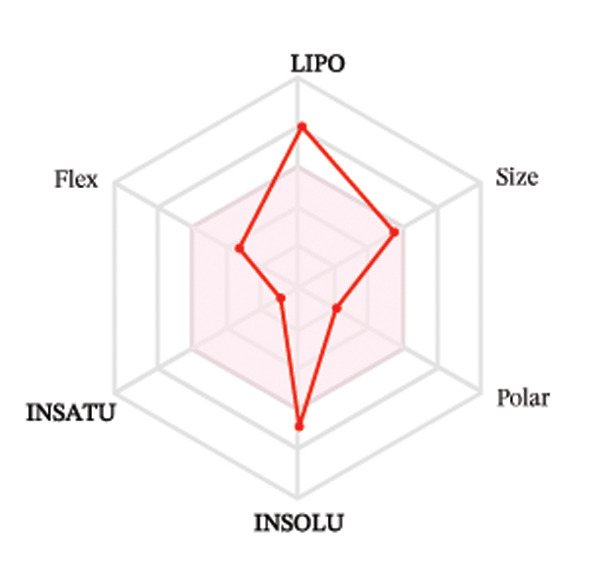
(l)

**Figure:**
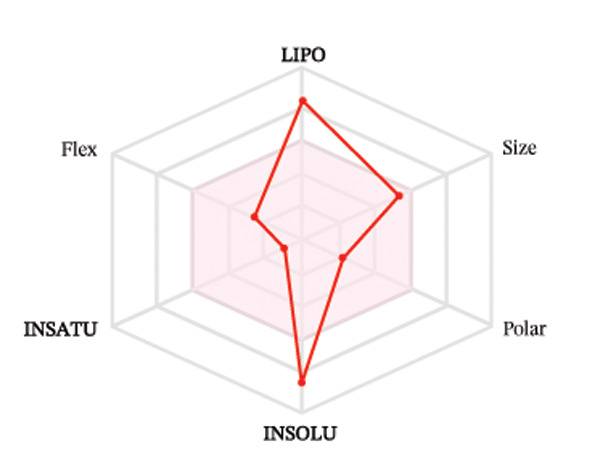
(m)

**Figure:**
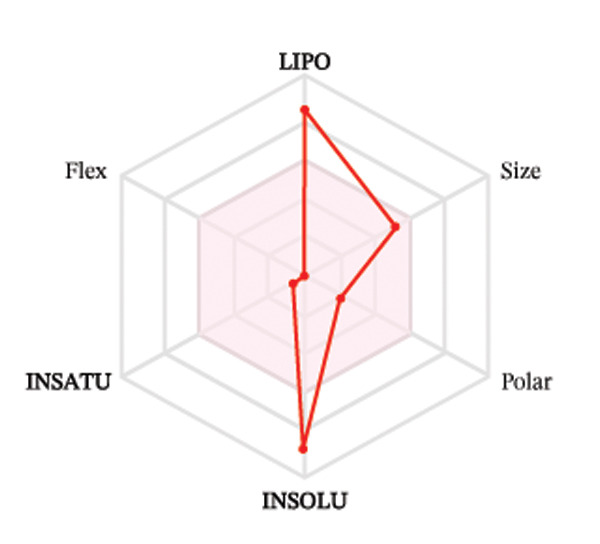
(n)

**Figure:**
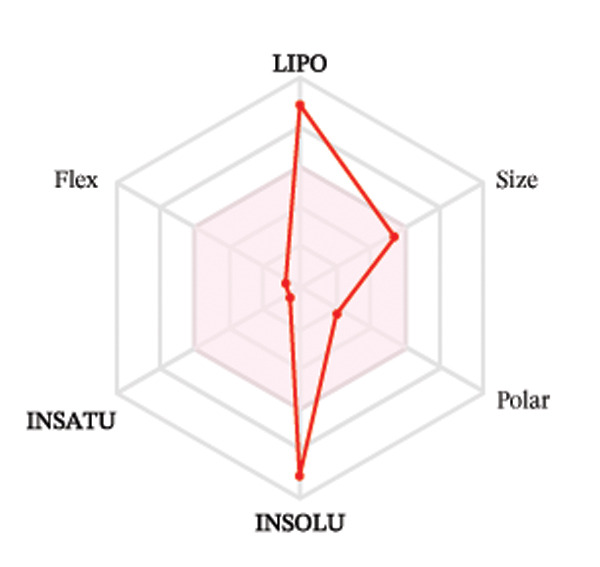
(o)

**Figure:**
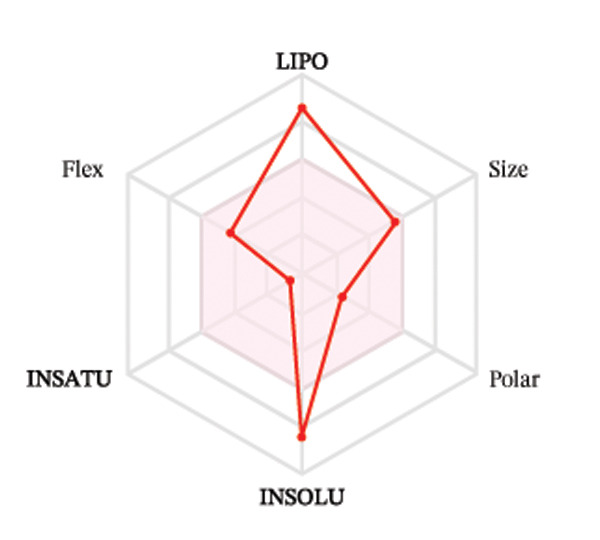
(p)

#### 3.3.3. Toxicity Evaluation

Evaluation of docked compounds with the best binding affinities showed that all compounds exhibited immunotoxicity, while toosendanin displayed immunotoxicity and cytotoxicity as well. It has been found that two compounds, toosendanin and dehydroergosterol, belong to Class III (50 < LD50 ≤ 300) and are found to be toxic if swallowed. Most of the compounds belonged to toxicity Class IV, indicating that they are harmful if swallowed in ranges (300 < LD50 ≤ 2000) (Table 9). Compounds such as 14b‐pregnan, olean‐12‐en‐3‐one, olean‐12‐EN‐3‐α‐YL acetate belonged to toxicity class V, meaning that they may be harmful (toxicity range: 2000 < LD50 ≤ 5000). Only one compound, β‐amyrin, belonged to Class VI and had an LD50 value greater than 5000. It means that it can be taken in greater than 5000 mg per kg of body mass. No compound belonged to toxicity Class I or II, which means that the fruit is not fatal immediately when swallowed, but can be toxic or can have adverse effects on the body or body organs.

**Table 9:** Toxicity evaluation of HEELS.

Compound name	Predicted LD_50_ (mg/kg)	Predicted toxicity class	Hepatotoxicity	Carcinogenicity	Mutagenicity	Immunotoxicity	Cytotoxicity
14b‐Pregnan	3660	5	−	−	−	+	−
Dehydroergosterol	288	3	−	−	−	+	−
Gamma‐sitosterol	890	4	−	−	−	+	−
5,6‐Dihydroergosterol	2000	4	−	−	−	+	−
Campesterol	890	4	−	−	−	+	−
(22E)‐Stigmasta‐5,22‐dien‐3‐ol	890	4	−	−	−	+	−
Ergost‐7‐en‐3‐ol, (3.β.,5.α.)‐	2000	4	−	−	−	+	−
9,19‐Cyclolanost‐7‐en‐3‐ol	2000	4	−	−	−	+	−
Chondrillasterol	2000	4	−	−	−	+	−
β‐Amyrin	70,000	6	−	−	−	+	−
Lanosterol	2000	4	−	−	−	+	−
5.α.‐Stigmast‐7‐en‐3.β.‐ol, (24S)‐	2000	4	−	−	−	+	−
Lupeol	2000	4	−	−	−	+	−
Olean‐12‐en‐3‐one	5000	5	−	−	−	+	−
Toosendanin	244	3	−	−	−	+	+
Olean‐12‐EN‐3‐α‐YL acetate	3460	5	−	+	−	+	−

*Note:* (+)—active and (−)—inactive.

## 4. Discussion

Phytochemical profiling is a vital approach for understanding the therapeutic potential of plant species, as it enables the identification of bioactive compounds that may be responsible for the pharmacological effects observed in traditional or modern medicinal applications [19]. In recent years, many plants have been studied for their antioxidant, antimicrobial, and anticancer properties, among others. *L. siceraria*, commonly known as bottle gourd, has been used in traditional medicine for its purported health benefits, but a comprehensive analysis of its bioactive compounds and therapeutic properties has been lacking. Our study aimed to fill this gap by conducting a detailed phytochemical profiling of the HEELS and examining its potential therapeutic applications. In our analysis, HEELS was found to be rich in several important phytochemicals, including carbohydrates, phenols, flavonoids, tannins, alkaloids, and cardiac glycosides. These compounds are often associated with antioxidant activity, which is believed to protect biological molecules from oxidative damage, a key factor in the development of various chronic diseases, including cancer and cardiovascular diseases. Our study found that the diverse phytochemical content of HEELS further supports its potential as a valuable source of natural antioxidants, which could be leveraged for health benefits.

The antioxidant properties of plant extracts have been well documented, particularly with respect to polyphenolic compounds like flavonoids and tannins. Phenolic compounds, especially those with high molecular weights, have been shown to exhibit strong antioxidant, anticancer, and antimutagenic properties [20]. Our findings confirmed this, showing a significant correlation between the concentration of phenolic compounds in HEELS and its antioxidant activity, as assessed by various assays, including DPPH, ABTS, FRAP, TAC, and NOS. Interestingly, while previous studies on *L. siceraria* have generally reported a higher phenolic content than flavonoids, our findings revealed that tannins were present in higher concentrations than both phenolic and flavonoid compounds in HEELS. This finding differs from those of other studies, which reported lower tannin levels than phenolic and flavonoid compounds [21, 22]. This discrepancy could be due to the differences in the plant parts used or variations in extraction methods, highlighting the complexity of phytochemical profiles in different extracts. Nevertheless, our study suggests that tannins may play a central role in the antioxidant and other therapeutic activities of HEELS.

To gain a deeper understanding of the chemical composition of HEELS, we employed GC‐MS, which identified 55 peaks corresponding to various bioactive compounds, including fatty acids, esters, terpenoids, alkanes, alkenes, and steroids. Among these, compounds such as n‐hexadecanoic acid, chondrillasterol, and hexadecanoic acid ethyl ester stood out as the major bioactive constituents. These compounds are well known for their antioxidant and anti‐inflammatory properties, which further support the biological activity observed in our study.

The urease inhibitory activity of *L. siceraria* was another focal point of this study. Urease, an enzyme produced by *Helicobacter pylori*, plays a crucial role in the pathogenesis of gastric ulcers and cancer. While conventional antibiotics are commonly used to treat *H. pylori* infections, a significant number of patients fail to respond to therapy. This has led to growing interest in exploring plant‐based alternatives for treating *H. pylori* infections [23]. Our study found that HEELS exhibited strong urease inhibitory activity, marking the first report of such an effect in *L. siceraria* fruit extract. This finding suggests that HEELS may serve as a promising natural alternative for managing gastric disorders associated with *H. pylori* infection.

Additionally, the ability to inhibit α‐amylase, an enzyme involved in carbohydrate digestion, is a well‐known strategy for managing hyperglycemia in diabetes. Several plant‐based compounds have been studied for their α‐amylase inhibitory potential, as they can delay carbohydrate digestion and absorption, leading to improved blood sugar control. In our study, the GC‐MS analysis revealed several bioactive compounds in HEELS, such as gamma‐sitosterol, campesterol, and lupeol, which exhibited strong binding affinities to α‐amylase. Our findings indicate that HEELS has a greater α‐amylase inhibitory effect compared to ethanolic seed extracts, supporting its potential use in diabetes management, as reported in similar studies [24, 25].

Tyrosinase inhibitors, on the other hand, are in high demand in the cosmetic industry, where they are used to reduce pigmentation and prevent skin aging. Traditional synthetic agents, such as hydroquinone, have been associated with adverse side effects, leading to increased interest in plant‐based alternatives with safer profiles [26]. In our study, HEELS exhibited strong tyrosinase inhibitory activity, which could make it a valuable ingredient in skin care formulations aimed at reducing pigmentation and aging signs. The bioactive compounds identified in the GC‐MS analysis, including β‐amyrin and lanosterol, demonstrated strong binding to tyrosinase, confirming their potential as natural tyrosinase inhibitors.

*L. siceraria* also demonstrated thrombolytic activity, which has drawn attention due to the growing interest in safer plant‐based alternatives for thrombolysis in treating blood clot–related conditions [27]. Our results showed that HEELS has thrombolytic potential, which aligns with earlier studies reporting such effects. Furthermore, our hemolysis assay revealed that the extract did not induce significant red blood cell rupture, suggesting that HEELS could be safely used for thrombolytic purposes, consistent with literature on its protective effects against hemolysis [28, 29].

The antibacterial properties of HEELS were assessed against both Gram‐positive and Gram‐negative bacterial strains. The extract showed dose‐dependent bactericidal activity, with higher concentrations leading to larger zones of inhibition. The presence of phytochemicals such as alkaloids, phenols, and flavonoids likely contributes to the antimicrobial properties observed in our study, as these compounds are often associated with antibacterial activity [30]. To further investigate the molecular mechanisms behind these biological activities, we employed in silico molecular docking studies.

The compounds identified in the GC‐MS analysis were docked against three key enzymes: tyrosinase, α‐amylase, and urease, to explore their potential as enzyme inhibitors. Several compounds from HEELS exhibited superior binding affinities to these enzymes compared to standard inhibitors such as kojic acid, acarbose, and thiourea, further supporting the idea that HEELS contains potent bioactive compounds capable of modulating these enzymes [31]. Finally, the pharmacokinetic properties of the top compounds identified through docking studies were evaluated using Lipinski’s rule of five, which predicts the oral bioavailability of potential drug candidates [32, 33]. Our findings showed that five compounds from HEELS met the criteria for good bioavailability, suggesting that these compounds could be suitable for oral administration in therapeutic applications [32, 34]. In terms of toxicity, the ProTox‐II tool [35] indicated that all compounds exhibited some degree of immunotoxicity, with one compound (β‐amyrin) showing low toxicity (LD_50_ > 5000 mg/kg). These findings suggest that while HEELS may be relatively safe, further studies are required to confirm its in vivo safety and therapeutic efficacy.

Overall, this study provides a comprehensive evaluation of the therapeutic potential of *L. siceraria* fruit, highlighting its antioxidant, antimicrobial, antidiabetic, and anti‐inflammatory properties, along with its promising applications in cosmetics and thrombolysis. The molecular docking and toxicity assessments offer valuable insights into the mechanisms of action and safety profile of HEELS, laying the foundation for future research on this plant as a natural therapeutic agent.

## 5. Conclusion

The findings of the recent study indicate that the bioactive phytoconstituents responsible for the pharmacological and therapeutic actions have been found in HEELS. HEELS demonstrated high TPC, TFC, TTC, and antioxidant capacity as well as strong α‐amylase, urease, and tyrosinase inhibition properties. Important phytocompounds were suggested to be present in the HEELS by GC‐MS analysis, which supported the compounds’ biological activity. Therefore, it can be said that HEELS has therapeutic potential in terms of antioxidant, antibacterial, thrombolytic, antihemolytic, enzyme inhibition, and antidiabetic properties, as demonstrated by in vitro investigation. Future in vivo studies are crucial to elucidate the precise mechanisms and identify the specific compounds responsible for the antioxidant, enzyme‐inhibiting, and thrombolytic activities of the selected plant, *L. siceraria*. Detailed investigations into the pharmacokinetics and pharmacodynamics will help establish the mechanisms through which these compounds exert their effects, thereby providing a foundation for potential therapeutic development and validating the plant’s traditional uses in medicine.

## Author Contributions

Hanan Y. Aati: project administration, writing–original draft, conceptualization, supervision, and resources. Renad Al‐Arifi: methodology and formal analysis. Chitchamai Ovatlarnporn: investigation, software, methodology, and project administration. Mohsin Abbas Khan: methodology and formal analysis. Abdul Rauf: writing–original draft and formal analysis. Hossam M. Hassan: formal analysis. Abdul Basit: methodology, investigation, writing–original draft, and software. Huma Rao: software and formal analysis. Laiba Rehman: data curation and software. Kashif ur Rehman Khan: supervision, investigation, writing–review and editing, and conceptualization.

## Funding

The authors thank Ongoing Research Funding Program (ORF‐2026‐504), King Saud University, Riyadh, Saudi Arabia.

## Disclosure

All the authors have read the manuscript and agree to submit it to Molecules for consideration.

## Conflicts of Interest

The authors declare no conflicts of interest.

## Supporting Information

The supporting information is available in a supporting file. S1. Pictorial illustration of diameter of the antibacterial effect of ELSF on three different bacterial strains.

S2. 2D and 3D interactions of tyrosinase with 14b‐pregnan, dehydroergosterol, gamma‐sitosterol, 5,6‐dihydroergosterol, campesterol (22E)‐Stigmasta‐5,22‐dien‐3‐ol, ergost‐7‐en‐3‐ol (3.β.,5.α.)‐, 9,19‐cyclolanost‐7‐en‐3‐ol, lanosterol, 5.α.‐Stigmast‐7‐en‐3.β.‐ol (24S)‐, toosendanin, olean‐12‐EN‐3‐α‐YL acetate, and kojic acid.

S3. 2D and 3D interactions of urease with 14b‐pregnan, dehydroergosterol, gamma‐sitosterol, 5,6‐dihydroergosterol, campesterol (22E)‐Stigmasta‐5,22‐dien‐3‐ol, ergost‐7‐en‐3‐ol (3.β.5.α.)‐, 9,19‐cyclolanost‐7‐en‐3‐ol, lanosterol, 5.α.‐Stigmast‐7‐en‐3‐β‐ol (24S)‐, toosendanin, olean‐12‐EN‐3‐α‐YL acetate, and thiourea.

S4. 2D and 3D interactions of alpha amylase with 14b‐pregnan, dehydroergosterol, gamma‐sitosterol, 5,6‐dihydroergosterol, campesterol (22E)‐Stigmasta‐5,22‐dien‐3‐ol, ergost‐7‐en‐3‐ol (3.β,5α)‐, 9,19‐cyclolanost‐7‐en‐3‐ol, lanosterol, 5.α.‐Stigmast‐7‐en‐3‐β‐ol (24S)‐, toosendanin, olean‐12‐EN‐3‐α‐YL acetate, and acarbose.

## Supporting information


**Supporting Information** Additional supporting information can be found online in the Supporting Information section.

## Data Availability

The data that support the findings of this study are available from the corresponding author upon reasonable request.
