# Supplementary material for: Bottle Gourd (Lagenaria siceraria): Novel Insights Into the Biochemical Propensities of the Unexplored Cultivar
Source: Scientifica (Cairo). 2026 Feb 23;2026:7463939. doi: 10.1155/sci5/7463939 (PMC12927919; doi:10.1155/sci5/7463939)
Supplement: Supplementary file 1 — Supporting Information Additional supporting information can be found online in the Supporting Information section. [file SCI5-2026-7463939-s001.docx]

**Bottle gourd (*Lagenaria siceraria*): Novel insights into the biochemical propensities of the unexplored cultivar**

**(Supplementary Data)**

**Supplementary Materials**

The supplementary data accompanying this manuscript provide additional experimental and computational evidence to support the study's findings. Supplementary Figure S1 presents a pictorial illustration of the antibacterial activity of the ethanolic leaf extract of *Lagenaria siceraria* (ELSF) against three bacterial strains, *Escherichia coli*, *Staphylococcus epidermidis*, and *Micrococcus luteus,* showing distinct inhibition zones that highlight the extract’s variable antibacterial efficacy. Supplementary Figures S2 to S4 depict detailed two- and three-dimensional molecular docking interactions between selected bioactive sterols and triterpenoids identified in *L. siceraria* and key target enzymes. Supplementary Figure S2 illustrates the docking conformations of compounds such as 14β-pregnan, dehydroergosterol, γ-sitosterol, campesterol, lanosterol, and toosendanin with the tyrosinase enzyme, demonstrating the active-site interactions responsible for their potential tyrosinase inhibitory effects. Supplementary Figure S3 presents the docking analysis of the same phytoconstituents with the urease enzyme, revealing hydrogen-bonding and hydrophobic interactions indicative of significant anti-urease potential. Supplementary Figure S4 presents the docking results of these compounds with the α-amylase enzyme, compared with the standard inhibitor acarbose, highlighting strong binding affinities that support their potential role in inhibiting α-amylase activity and suggesting antidiabetic relevance. Together, these supplementary materials provide visual and structural insights that reinforce the biochemical and pharmacological interpretations discussed in the main manuscript.

| 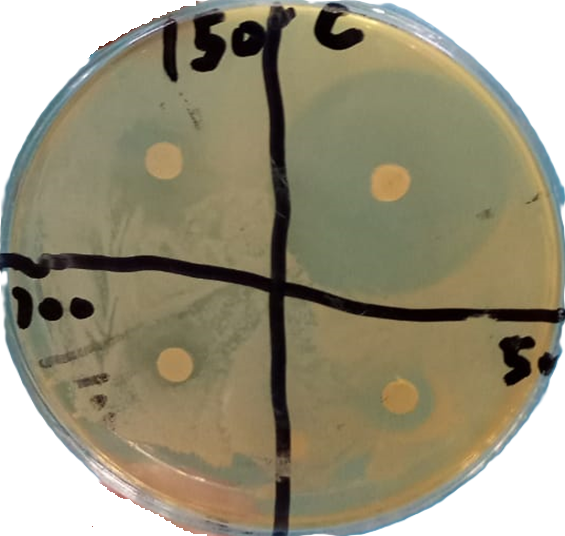  **Escherichia coli** | 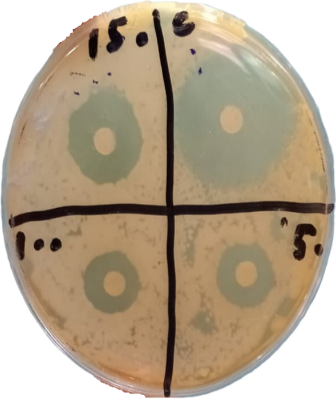  **Staphylococcus epidermidis** |
| --- | --- |
| 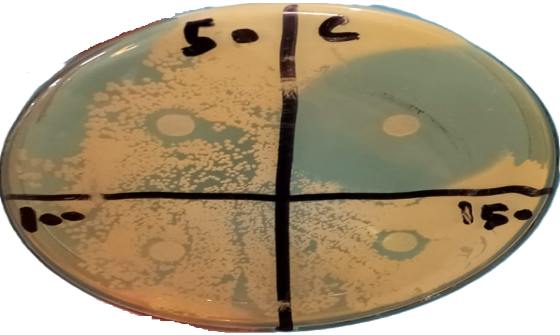  **Micrococcus luteus** | |

**S1.** Pictorial illustration of diameter of antibacterial effect of ELSF on different three bacterial strains

| 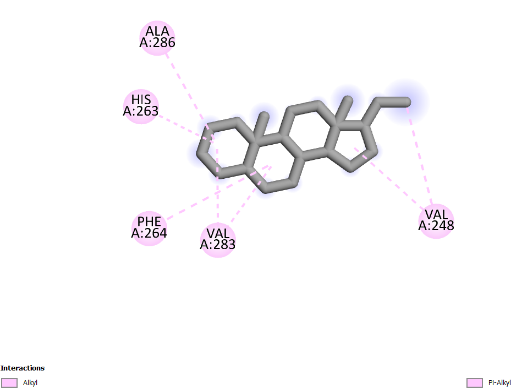 | | | | 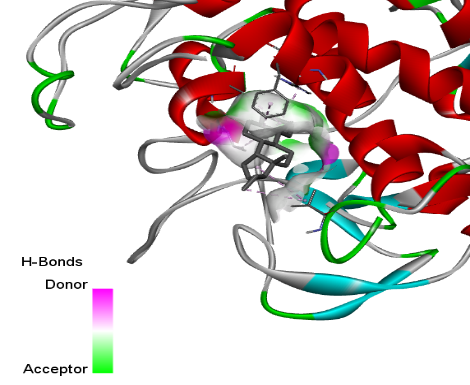 |
| --- | --- | --- | --- | --- |
| 2d and 3d structure of 14b-pregnan with tyrosinase enzyme | | | | |
| 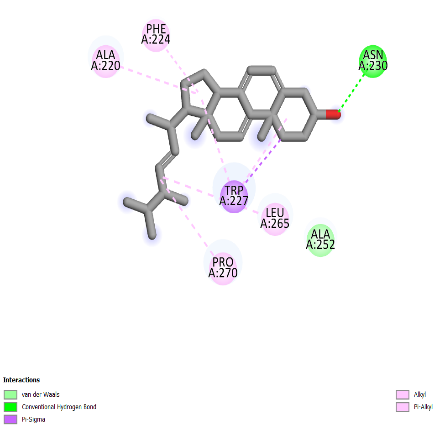 | | | | 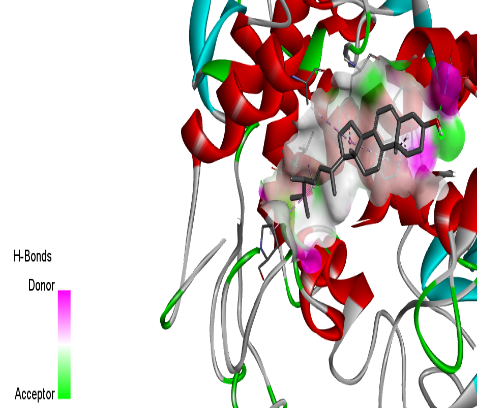 |
| 2d and 3d structure of Dehydroergosterol with tyrosinase enzyme | | | | |
| 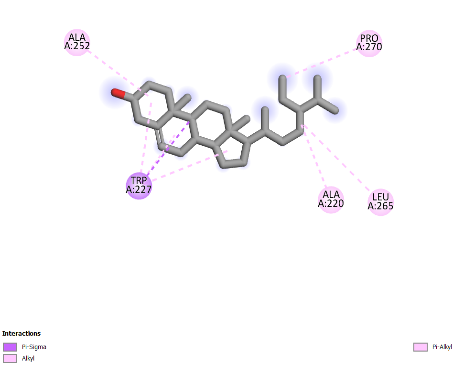 | 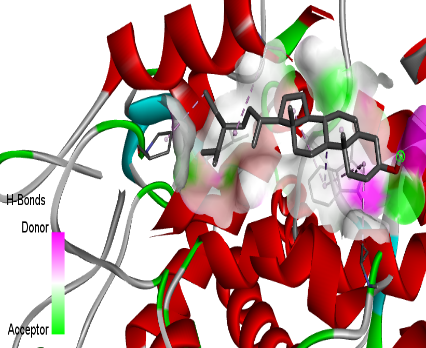 | | | |
| 2d and 3d structure Gamma sitosterol of with tyrosinase enzyme | | | | |
| 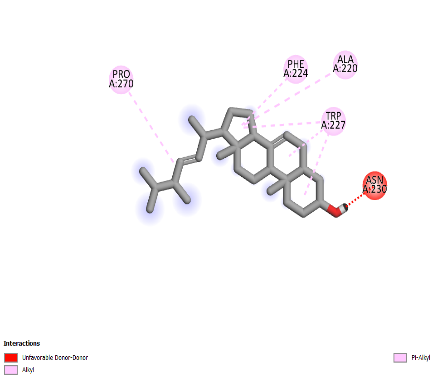 | 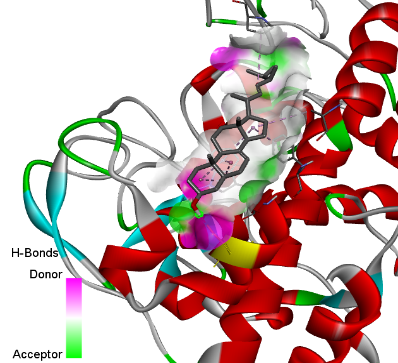 | | | |
| 2d and 3d structure of 5,6-Dihydroergosterol with tyrosinase enzyme | | | | |
| 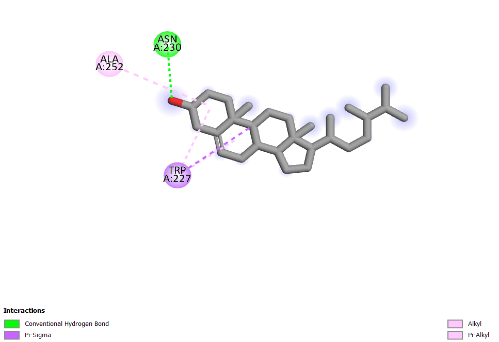 | 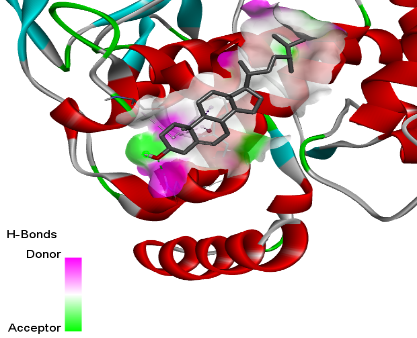 | | | |
| 2d and 3d structure of Campesterol with tyrosinase enzyme | | | | |
| 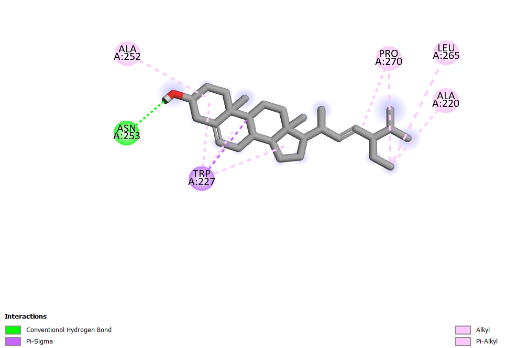 | 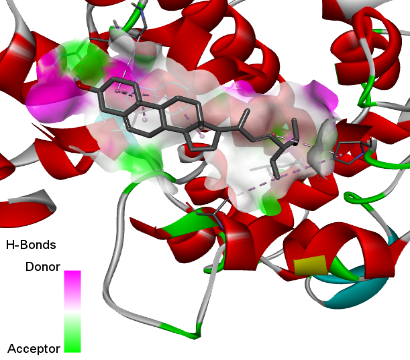 | | | |
| 2d and 3d structure of (22E)-Stigmasta-5,22-Dien-3-ol with tyrosinase enzyme | | | | |
| 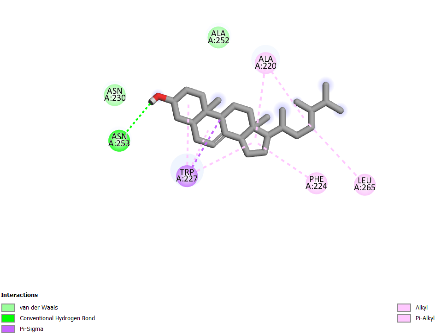 | 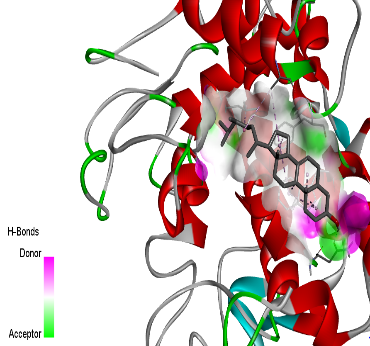 | | | |
| 2d and 3d structure of Ergost-7-en-3-ol, (3.β.,5.α.)- with tyrosinase enzyme | | | | |
| 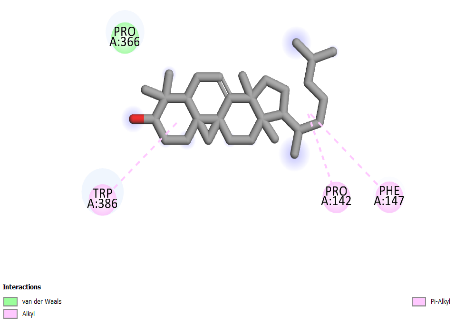 | 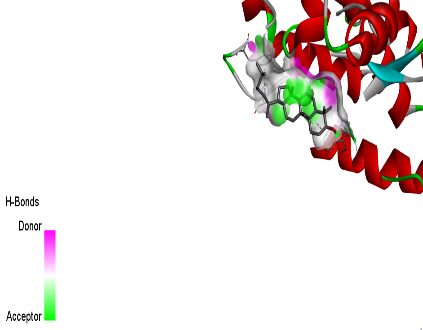 | | | |
| 2d and 3d structure of 9,19-Cyclolanost-7-en-3-ol with tyrosinase enzyme | | | | |
| 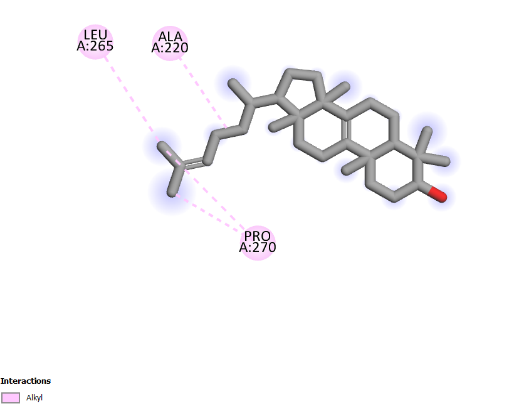 | 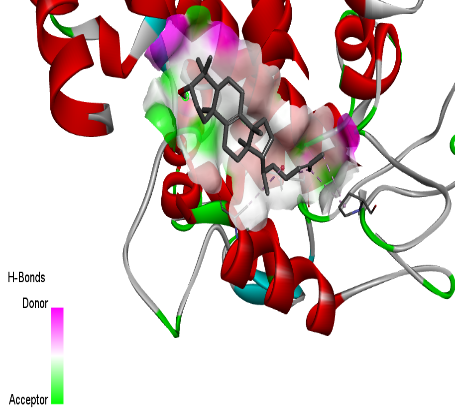 | | | |
| 2d and 3d structure of Lanosterol with tyrosinase enzyme | | | | |
| 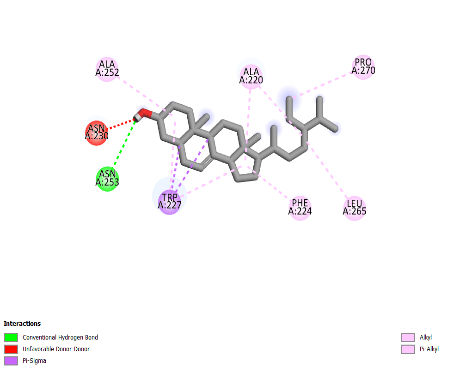 | 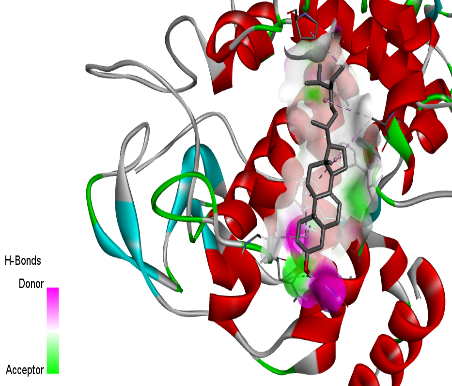 | | | |
| 2d and 3d structure of 5.α.-Stigmast-7-en-3.β.-ol, (24S) with tyrosinase enzyme | | | | |
| 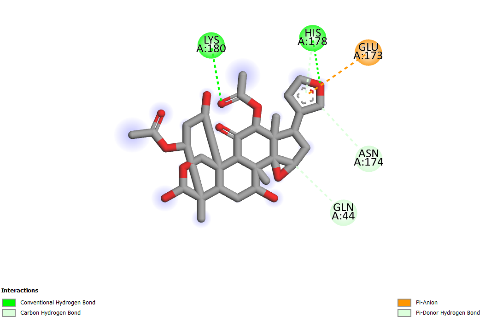 | | 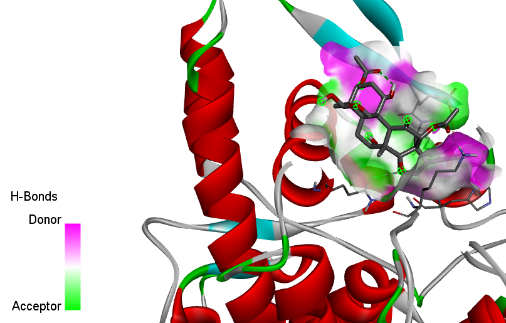 | | |
| 2d and 3d structure of Toosendanin with tyrosinase enzyme | | | | |
| 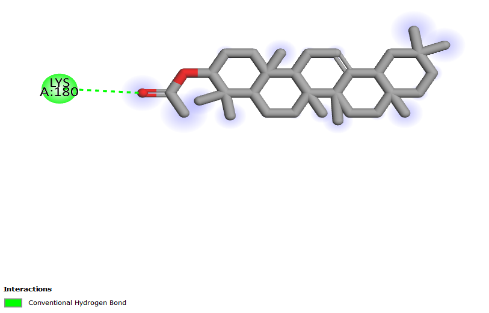 | | 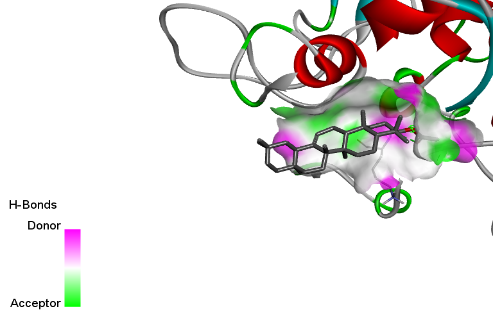 | | |
| 2d and 3d structure of Olean-12-EN-3-α-YL acetate with tyrosinase enzyme | | | | |
| 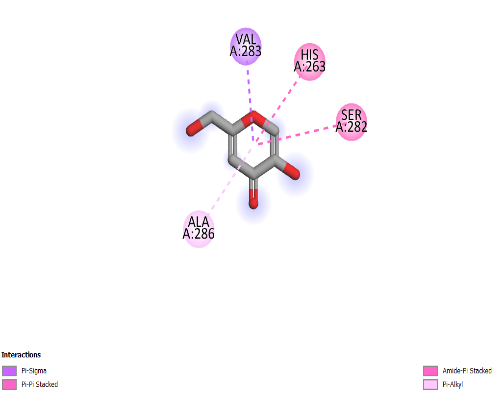 | | | 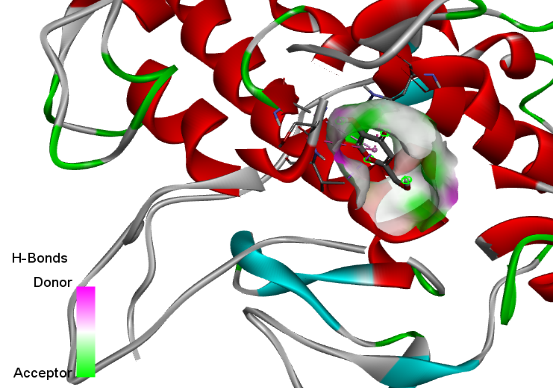 | |
| 2d and 3d structure of kojic acid with tyrosinase enzyme | | | | |

**S2 .** 2-D and 3-D interaction of tyrosinase with 14b-pregnan, Dehydroergosterol, Gamma sitosterol, 5,6-Dihydroergosterol, Campesterol, (22E)-Stigmasta-5,22-Dien-3-ol, Ergost-7-en-3-ol, (3.β.,5.α.)-, 9,19-Cyclolanost-7-en-3-ol, Lanosterol, 5.α.-Stigmast-7-en-3.β.-ol, (24S)-, Toosendanin, Olean-12-EN-3-α-YL acetate and kojic acid.

| 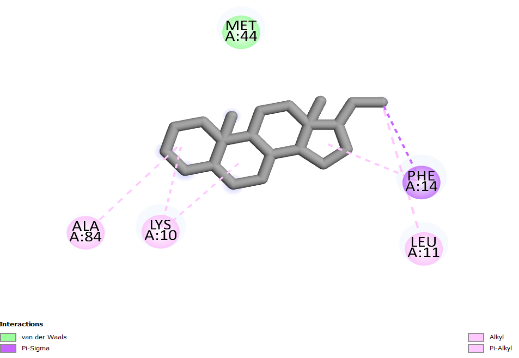 | | 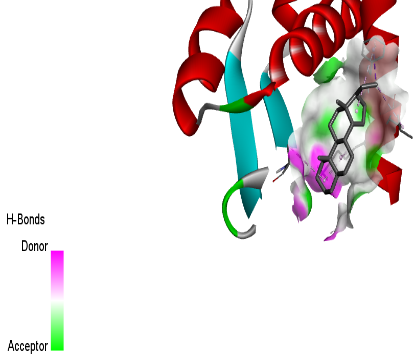 | |
| --- | --- | --- | --- |
| 2d and 3d structure of 14b-pregnan with urease enzyme | | | |
| 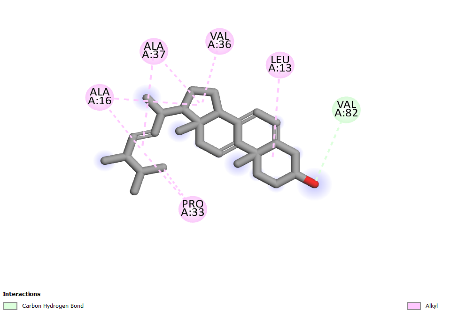 | | 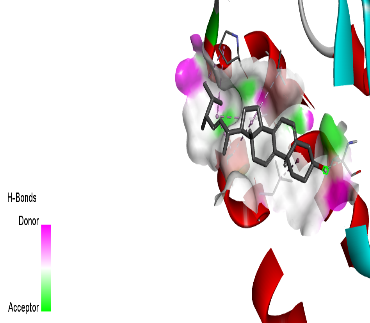 | |
| 2d and 3d structure of Dehydroergosterol with urease enzyme | | | |
| 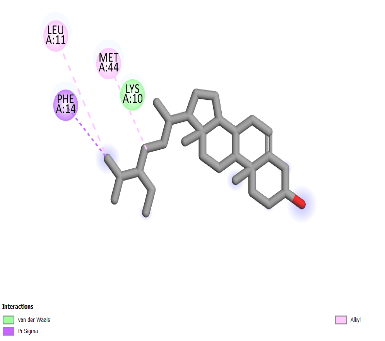 | | 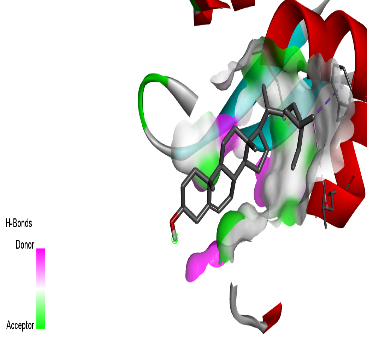 | |
| 2d and 3d structure of Gamma sitosterol with urease enzyme | | | |
| 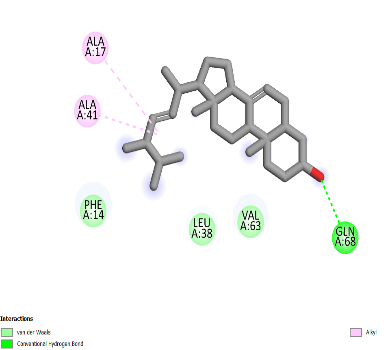 | | 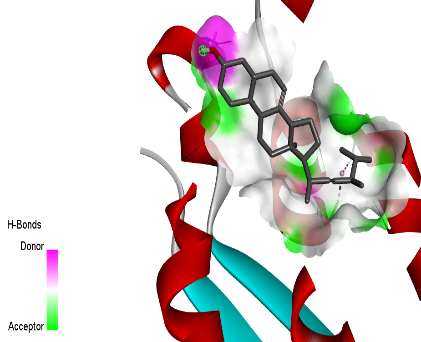 | |
| 2d and 3d structure of 5,6-Dihydroergosterol with urease enzyme | | | |
| 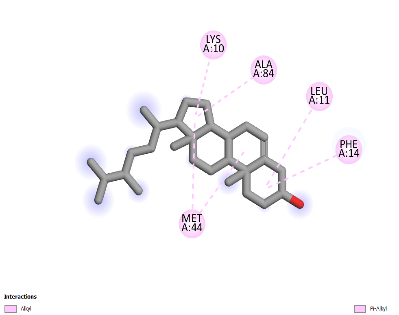 | | | 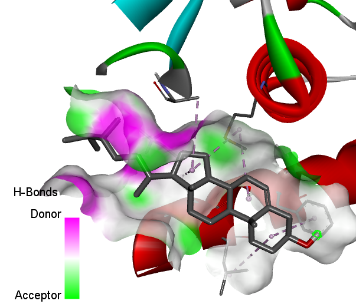 |
| 2d and 3d structure of Campesterol with urease enzyme | | | |
| 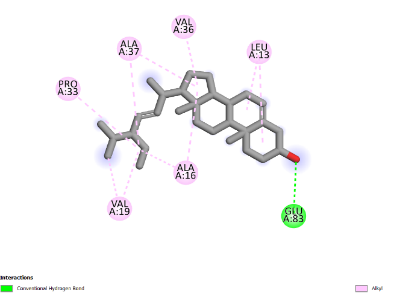 | | | 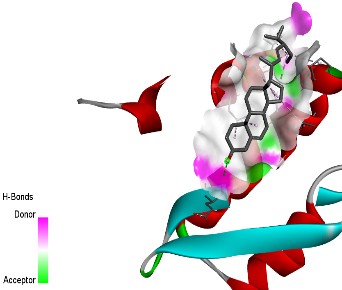 |
| 2d and 3d structure of (22E)-Stigmasta-5,22-Dien-3-ol with urease enzyme | | | |
| 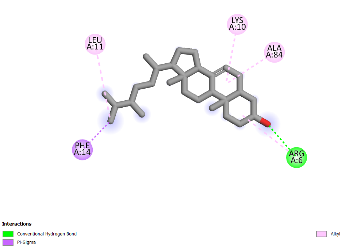 | | | 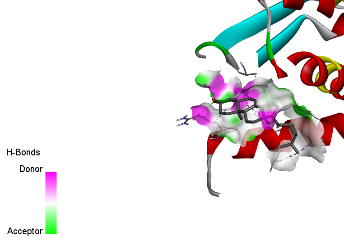 |
| 2d and 3d structure Ergost-7-en-3-ol, (3.β.,5.α.)- of with urease enzyme | | | |
| 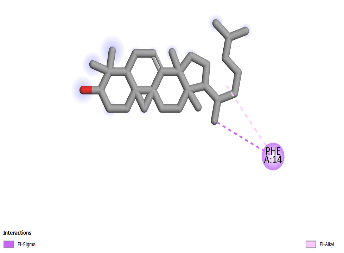 | | 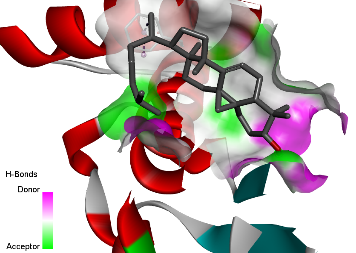 | |
| 2d and 3d structure of 9,19-Cyclolanost-7-en-3-ol with urease enzyme | | | |
| 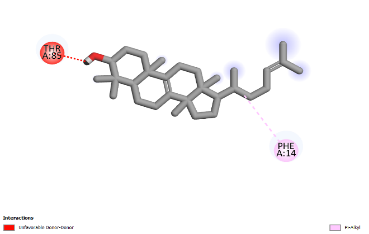 | | 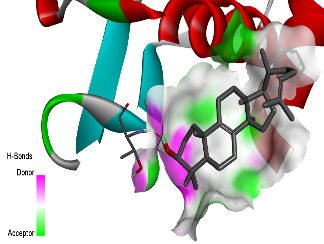 | |
| 2d and 3d structure of Lanosterol with urease enzyme | | | |
| 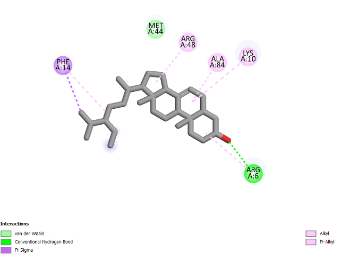 | | 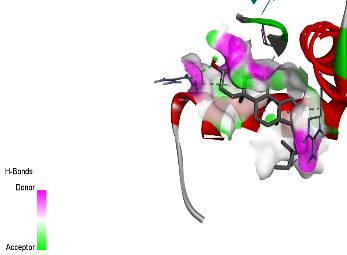 | |
| 2d and 3d structure of 5.α.-Stigmast-7-en-3-β-ol, (24S)- with urease enzyme | | | |
| 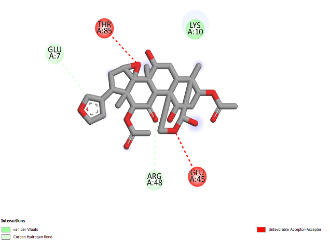 | 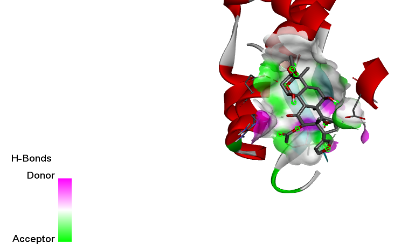 | | |
| 2d and 3d structure of Toosendanin with urease enzyme | | | |
| 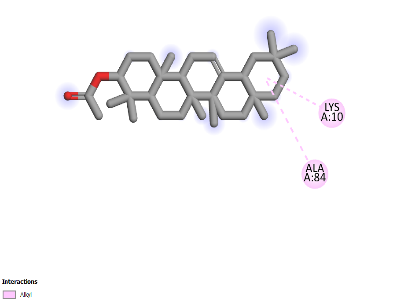 | 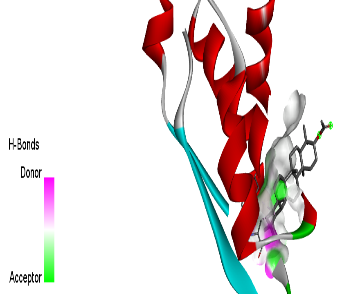 | | |
| 2d and 3d structure of Olean-12-EN-3-α-YL acetate with urease enzyme | | | |
| 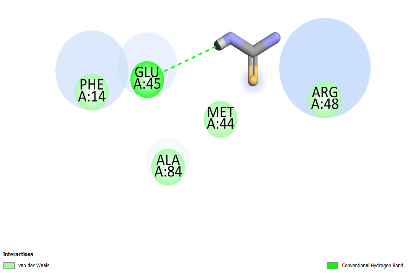 | 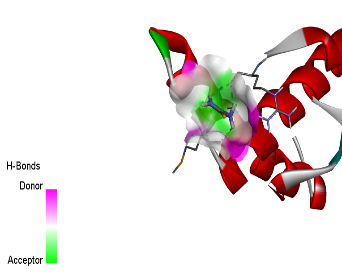 | | |
| 2d and 3d structure of thiourea with urease enzyme | | | |

**S3.** 2-D and 3-D interaction of urease with 14b-pregnan, Dehydroergosterol, Gamma sitosterol, 5,6-Dihydroergosterol, Campesterol, (22E)-Stigmasta-5,22-Dien-3-ol, Ergost-7-en-3-ol, (3.β.5.α.)-, 9,19-Cyclolanost-7-en-3-ol, Lanosterol, 5.α.-Stigmast-7-en-3-β-ol, (24S)-, Toosendanin, Olean-12-EN-3-α-YL acetate and thiourea.

| 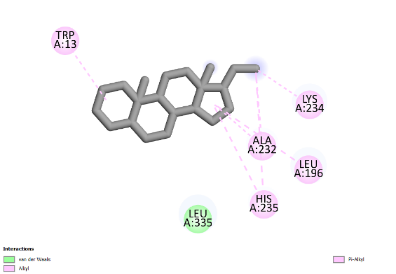 | 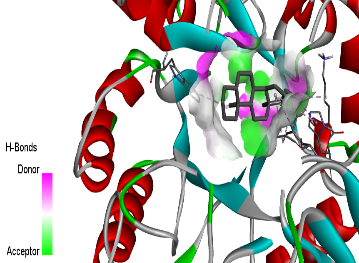 | | |
| --- | --- | --- | --- |
| 2d and 3d structure of 14b-pregnan with α amylase enzyme | | | |
| 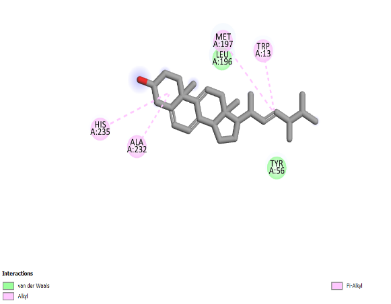 | 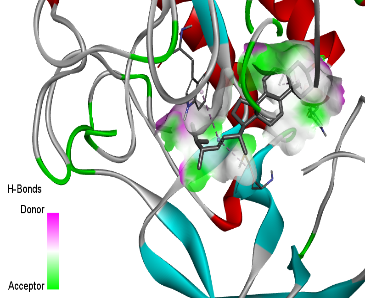 | | |
| 2d and 3d structure of Dehydroergosterol with α amylase enzyme | | | |
| 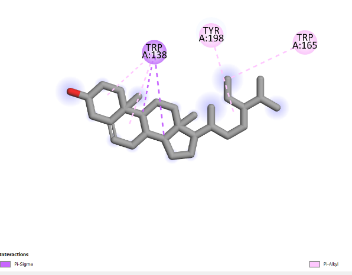 | 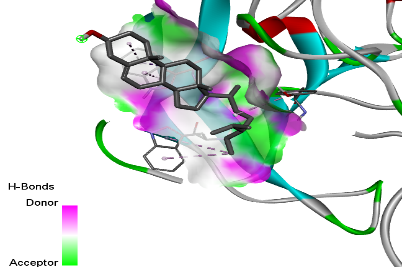 | | |
| 2d and 3d structure of Gamma sitosterol with α amylase enzyme | | | |
| 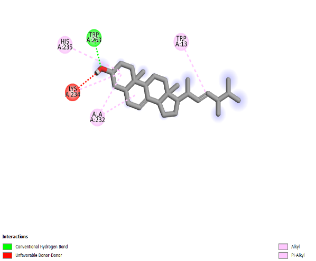 | 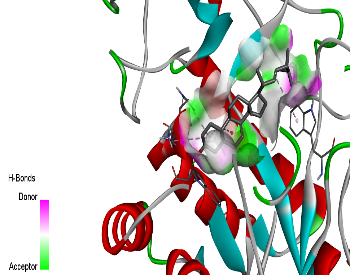 | | |
| 2d and 3d structure of 5,6-Dihydroergosterol with α amylase enzyme | | | |
| 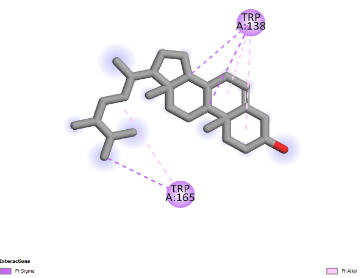 | | 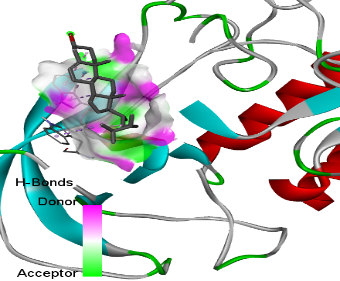 | |
| 2d and 3d structure of Campesterol with α amylase enzyme | | | |
| 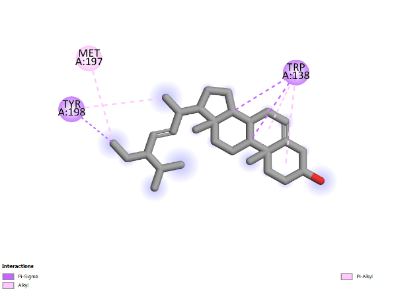 | | 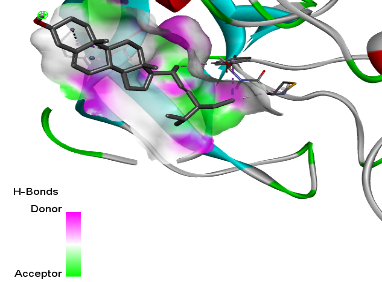 | |
| 2d and 3d structure of (22E)-Stigmasta-5,22-Dien-3-ol with α amylase enzyme | | | |
| 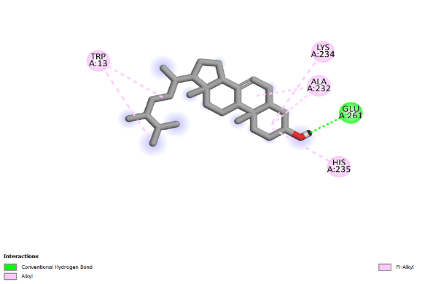 | | | 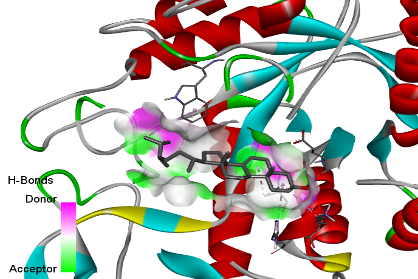 |
| 2d and 3d structure of Ergost-7-en-3-ol, (3.β.,5.α.)- with α amylase enzyme | | | |
| 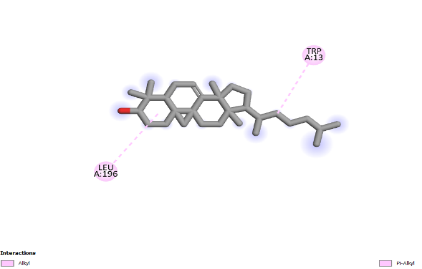 | | | 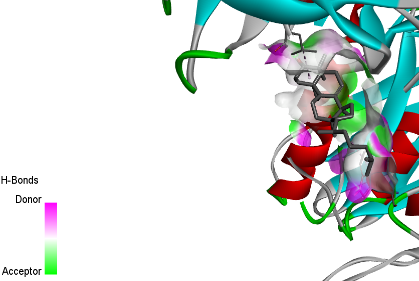 |
| 2d and 3d structure of 9,19-Cyclolanost-7-en-3-ol with α amylase enzyme | | | |
| 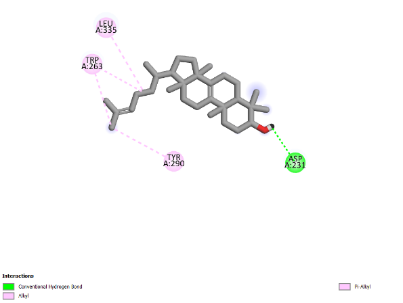 | | | 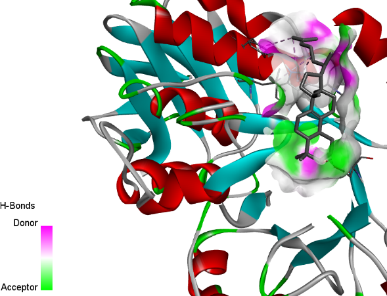 |
| 2d and 3d structure of Lanosterol with α amylase enzyme | | | |
| 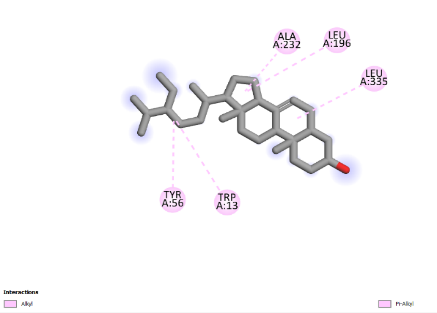 | | | 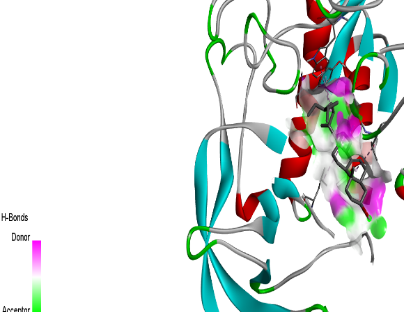 |
| 2d and 3d structure of 5.α.-Stigmast-7-en-3.β.-ol, (24S)- with α amylase enzyme | | | |
| 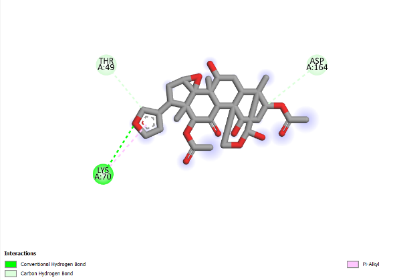 | | | 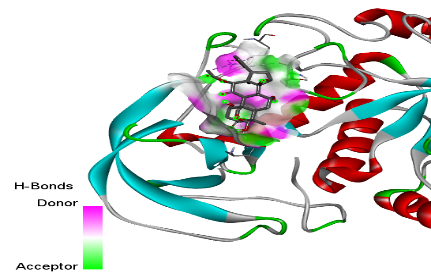 |
| 2d and 3d structure of Toosendanin with α amylase enzyme | | | |
| 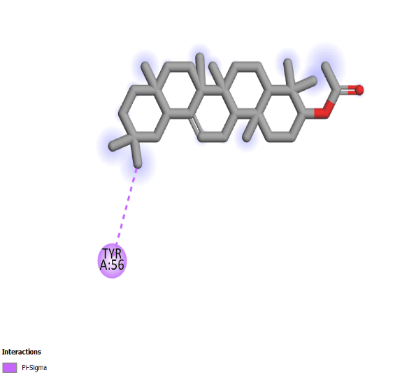 | | | 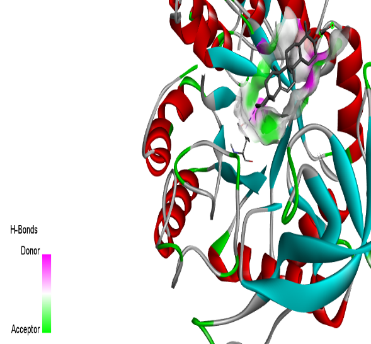 |
| 2d and 3d structure of Olean-12-EN-3-alpha-YL acetate with alpha amylase enzyme | | | |
| 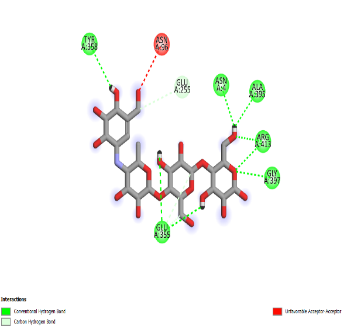 | | | 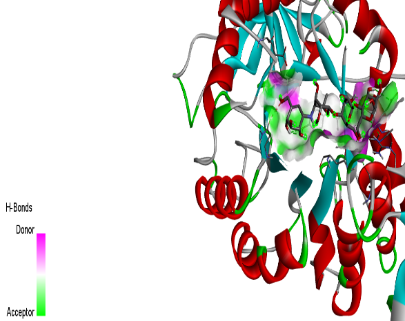 |
| 2d and 3d structure of Acarbose with alpha amylase enzyme | | | |

**S4.** 2-D and 3-D interaction of alpha amylase with 14b-pregnan, Dehydroergosterol, Gamma sitosterol, 5,6-Dihydroergosterol, Campesterol, (22E)-Stigmasta-5,22-Dien-3-ol, Ergost-7-en-3-ol, (3.β,5α)-, 9,19-Cyclolanost-7-en-3-ol, Lanosterol, 5.α.-Stigmast-7-en-3-β-ol, (24S)-, Toosendanin, Olean-12-EN-3-α-YL acetate and Acarbose.
